# Broad Blockade Antibody Responses in Human Volunteers after Immunization with a Multivalent Norovirus VLP Candidate Vaccine: Immunological Analyses from a Phase I Clinical Trial

**DOI:** 10.1371/journal.pmed.1001807

**Published:** 2015-03-24

**Authors:** Lisa C. Lindesmith, Martin T. Ferris, Clancy W. Mullan, Jennifer Ferreira, Kari Debbink, Jesica Swanstrom, Charles Richardson, Robert R. Goodwin, Frank Baehner, Paul M. Mendelman, Robert F. Bargatze, Ralph S. Baric

**Affiliations:** 1 Department of Epidemiology, University of North Carolina, Chapel Hill, North Carolina, United States of America; 2 Department of Genetics, University of North Carolina, Chapel Hill, North Carolina, United States of America; 3 The EMMES Corporation, Rockville, Maryland, United States of America; 4 Takeda Vaccines, Deerfield, Illinois, United States of America; 5 Takeda Pharmaceutical International, Zurich, Switzerland; CDC, UNITED STATES

## Abstract

**Background:**

Human noroviruses (NoVs) are the primary cause of acute gastroenteritis and are characterized by antigenic variation between genogroups and genotypes and antigenic drift of strains within the predominant GII.4 genotype. In the context of this diversity, an effective NoV vaccine must elicit broadly protective immunity. We used an antibody (Ab) binding blockade assay to measure the potential cross-strain protection provided by a multivalent NoV virus-like particle (VLP) candidate vaccine in human volunteers.

**Methods and Findings:**

Sera from ten human volunteers immunized with a multivalent NoV VLP vaccine (genotypes GI.1/GII.4) were analyzed for IgG and Ab blockade of VLP interaction with carbohydrate ligand, a potential correlate of protective immunity to NoV infection and illness. Immunization resulted in rapid rises in IgG and blockade Ab titers against both vaccine components and additional VLPs representing diverse strains and genotypes not represented in the vaccine. Importantly, vaccination induced blockade Ab to two novel GII.4 strains not in circulation at the time of vaccination or sample collection. GII.4 cross-reactive blockade Ab titers were more potent than responses against non-GII.4 VLPs, suggesting that previous exposure history to this dominant circulating genotype may impact the vaccine Ab response. Further, antigenic cartography indicated that vaccination preferentially activated preexisting Ab responses to epitopes associated with GII.4.1997. Study interpretations may be limited by the relevance of the surrogate neutralization assay and the number of immunized participants evaluated.

**Conclusions:**

Vaccination with a multivalent NoV VLP vaccine induces a broadly blocking Ab response to multiple epitopes within vaccine and non-vaccine NoV strains and to novel antigenic variants not yet circulating at the time of vaccination. These data reveal new information about complex NoV immune responses to both natural exposure and to vaccination, and support the potential feasibility of an efficacious multivalent NoV VLP vaccine for future use in human populations.

**Trial Registration:**

ClinicalTrials.gov NCT01168401

## Abstract 2

Lisa Lindesmith and colleagues assess the potential of a candidate virus-like particle (VLP) vaccine to induce antibody responses to antigenically divergent norovirus strains.

## Editors' Summary

**Background:**

Worldwide, noroviruses cause one in five cases of viral gastroenteritis (often called stomach flu or winter vomiting disease), the symptoms of which include nausea, vomiting, and diarrhea. There is no specific treatment for infection with these highly contagious viruses, and no established approach to vaccine development. While most people recover from the symptoms of norovirus infection within a few days, young children and the elderly may become severely ill or die. An estimated annual 300 million cases of norovirus infection contribute to roughly 260,000 deaths, mostly among this vulnerable demographic and mostly in low-income countries. Like influenza viruses, many noroviruses are evolving via a process known as antigenic drift. Antigens are components of infectious agents (including viruses) that are recognized by antibodies, proteins that bind to and neutralize foreign invaders. Over time, noroviruses develop small changes in their antigens that allow them to escape from antibodies produced in response to earlier infections. Every two to four years, because of accumulated antigenic drift, a new strain of norovirus emerges to which the human population has no direct antibody immunity, and an outbreak occurs. Because vaccines usually contain a component of the infectious agent that stimulates immunity, antigenic drift complicates the process of vaccine development. To be worth the cost and effort, a norovirus vaccine must confer immunity against a diverse range of norovirus strains, ideally including strains beyond those represented within the vaccine itself.

Partly because there is not a reliable method for growing noroviruses in the laboratory, recent efforts have focused on developing candidate vaccines using virus-like particles (VLPs). VLPs are constructed from laboratory-generated molecules of the virus’s capsid (outer shell). These capsid proteins self-assemble into icosahedral VLPs, which resemble the viral shell. VLPs cannot infect people or cause illness, but because they contain viral antigens, they can induce the immune system to produce antibodies that may neutralize actual viruses. VLPs can also be used to study the antibodies that people produce in response to vaccination or infection.

**Why Was This Study Done?:**

VLP-based vaccines are relatively new, and their capacity to elicit a broad immune response conferring protection to an evolving range of norovirus strains is not established. One VLP vaccine based on a single strain that circulates primarily in children conferred immunity to that strain. Another, multivalent (containing a mix of VLPs from more than one strain) VLP vaccine elicited antibody generation, but in a phase I clinical trial did not confer immunity to infection by a strain that had previously circulated globally. In the current study, the researchers explored two key questions using laboratory analysis of blood samples drawn from participants in that trial. First, they tested whether the vaccine elicits antibody responses to a broad range of norovirus strains, as antibody responses can provide clues to the potential for this type of vaccine to confer broad immunity in the future. Second, they investigated how preexisting exposure to noroviruses affects the immune system’s response to a vaccine—strategic information that could aid in future vaccine development.

**What Did the Researchers Do and Find?:**

The researchers tested serum (blood without cells or clotting proteins; serum contains the antibodies generated by the immune system) collected from ten participants receiving one injection of the VLP vaccine followed by a second injection 28 days later. They analyzed the serum specimens for antibodies to vaccine VLPs and also to VLPs representing viruses that were not contained in the vaccine. They used two methods, both utilizing VLPs generated from 11 norovirus strains: a traditional method that assesses binding of serum antibodies to each of these VLPs, and a more recent method that assays the ability of antibodies to block the interaction of each VLP with a molecule on intestinal cells that binds to the virus (the gut epithelial ligand), enabling norovirus to enter and infect cells. Prior studies suggest that this latter assay may be a better proxy for actual immunity.

The researchers’ major finding is that a multivalent VLP vaccine (two VLPs representing four strains of norovirus: one from a subgroup called genotype GI.1 and another consensus VLP of three strains from the subgroup GII.4) can rapidly elicit serum antibodies that bind a range of vaccine and non-vaccine VLPs, and that block binding of these VLPs to the gut epithelial ligand. Notably, vaccine recipients also generated antibodies reactive to two novel VLPs representing human noroviruses that they could not have previously encountered, indicating that prior exposure to each norovirus strain was not required for the full antibody response following vaccination. However, based on an analysis of which specific epitopes (small regions on an antigen) the population of antibodies binds, the authors report that antibody responses to the vaccine prominently target epitopes of a 1997 strain of human GII.4 norovirus, and propose that exposure history does influence the antibody response.

**What Do these Findings Mean?:**

These findings raise the possibility that the VLP vaccine may induce immunity not only to norovirus strains that have caused past outbreaks, but also to variants that have yet to enter the population—a necessary attribute given the antigenic drift observed among noroviruses. The study also indicates that VLP-induced antibody responses to norovirus are consistent with the “antigenic seniority” model, in which strains to which an individual was previously exposed influence the binding properties of a vaccine-induced antibody population. This latter finding may influence the design of future norovirus vaccines.

These results must be interpreted cautiously, particularly as they pertain to the potential for a norovirus vaccine to protect against natural infection. The study is small, and antibody binding and blocking assays may not replicate how the immune system of a vaccine recipient will respond to true norovirus infection. Additionally, the study participants were all adults aged 18 to 49 years, while a vaccine is most needed for young children (who account for the majority of severe infections) and the elderly (who are most likely to die from infection). Unlike the study participants, young children lack preexisting antibodies to norovirus. Older people are more likely to have been previously exposed to norovirus, but may show attenuated immune responses to vaccination. Adapting to the different immune responses of these two groups remains a central challenge to norovirus vaccine development.

**Additional Information:**

Please access these websites via the online version of this summary at http://dx.doi.org/10.1371/journal.pmed.1001807.

- The World Health Organization provides a comprehensive description of the disease burden from diarrheal disease
- The MedlinePlus encyclopedia has a page on viral gastroenteritis (in English and Spanish)
- The US Centers for Disease Control and Prevention provides information on disease trends and outbreaks
- The US Department of Health and Human Services offers guidance for prevention based on food safety
- A 2014 interview with Academic Editor Benjamin Lopman explores the difficulty of developing a norovirus vaccine
- The authors have previously published findings on the evolution of norovirus strains in *PLOS Medicine* and have discussed the challenges of norovirus therapeutic design in *PLOS Pathogens*

## Introduction

Diarrheal disease is a significant global health problem with an estimated 1.7 billion annual cases. Symptoms from infection range from dehydration to malnutrition and even to early death, particularly among children less than 5 y old and older adults [1]. Norovirus (NoV) strains are responsible for about 50% of global outbreaks of gastroenteritis and cause an estimated 21 million infections per year in the United States alone [2]. Although most NoV infections result in illness of modest severity, infection in children, older adults, immune-compromised individuals, and individuals with underlying medical conditions such as malnutrition can have severe and even fatal consequences [3–13]. In addition to benefitting these particularly vulnerable populations, an effective NoV vaccine would also benefit members of the military, travelers, and childcare, healthcare, and food industry workers.

NoVs are positive-sense, single-stranded RNA viruses in the Caliciviridae family, with genogroups GI and GII causing almost all human infections. Although new approaches to classification have been suggested [14], NoV genotyping is based primarily on the amino acid sequence of the major capsid protein (VP1) encoded by ORF 2 [15]. The genogroups are further subdivided into a total of 30 different known genotypes. The GII.4 NoV strains cause 70%–80% of documented NoV outbreaks and account for the largest percentage of long-term-care-facility- and vacation-related (e.g., cruise ship) outbreaks [2]. GII strains other than GII.4 cause more food-service- and community-related outbreaks than the less frequent GI strains [2], though both GI and GII strains are routinely detected in outbreak investigations.

NoVs are non-enveloped, small, round, structured viruses. Expression of the ORF 2 sequence results in abundant capsid protein production. These proteins self-assemble into icosahedral virus-like particles (VLPs) that are morphologically and antigenically indistinguishable from native virions [16,17]. The NoV major capsid protein is divided into the shell and protruding domains. The surface-exposed P2 subdomain (residues 279–405) [17] interacts with histoblood group antigens (HBGAs) expressed on gut epithelial cells, facilitating virus binding and entry resulting in infection [18]. Gut HBGA expression is largely dependent upon a functional secretor enzyme (secretor-positive phenotype). Individuals lacking a functional secretor enzyme are secretor-negative and at reduced risk of NoV infection. In the absence of a robust cell culture or small animal model, NoV VLPs serve as surrogate viruses in immunological and biochemical assays, including an in vitro assay of antibody (Ab) blockade of VLP–HBGA interaction. The blockade assay is useful in identifying Abs that functionally block binding of NoV capsid protein to carbohydrate ligands; these Abs are a subset of serum Abs that bind to the VLPs as identified by enzyme immunoassay (EIA) [19,20]. The blockade assay has been investigated for clinical relevance and holds promise as a surrogate neutralization assay, as shown in both infected chimpanzees [21] and Norwalk-virus-challenged human participants as a component of protective immunity [22,23].

**Fig 1:**
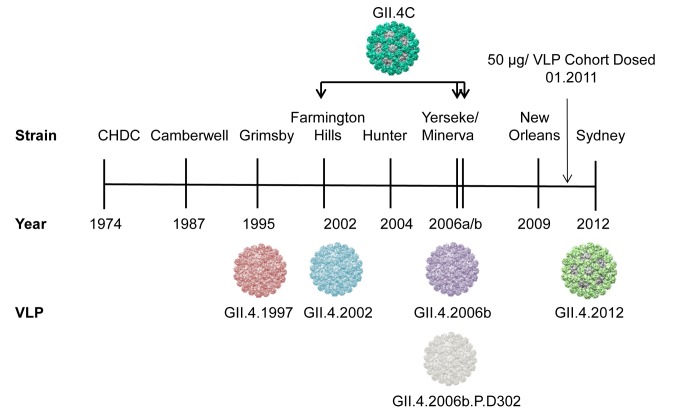
Temporal relationship between epidemiologically important GII.4 strains, relative to the virus-like particles and sera used in this study. The vaccine GII.4 component (GII.4C) is a consensus VLP composed of GII.4.2002, GII.4.2006a, and GII.4.2006b sequences. GII.4.2006b.P.D302 represents a strain that evolved in vivo and was isolated from an immune-compromised person.

The ORF 2 gene of GII.4 strains is evolving via epochal evolution, a pattern of change characterized by circulating strain replacement with a newly emergent strain, followed by a period of relative stasis [19,24]. Resulting new predominant GII.4 NoV strains (see Fig. 1) can have altered antigenicity and potentially altered ligand binding profiles [19]. Within the major capsid protein gene of GII.4 strains, residues of the P2 subdomain are under selective pressure by the host immune response; this pressure is one potential mechanism that drives viral evolution, resulting in strain antigenic drift and escape from herd immunity [19,25–28]. We have identified three GII.4-specific, evolving blockade Ab epitopes. Epitope A is hypervariable, and loss of epitope A blockade Abs correlates with viral escape from herd immunity [20,25,27,29]. Epitope D is less variable and, importantly, also modulates the ligand binding properties of GII.4 strains, explaining altered HBGA binding patterns in evolving strains [19,25,30]. Epitope E is a confirmed Farmington Hills–specific blockade epitope [31]. Epitope F is conserved across all GII.4 strains measured to date. The amino acid coordinates of epitope F are not yet known, but we have both human and mouse monoclonal antibodies (mAbs) that recognize this epitope and have identified a domain (the NERK motif) within the capsid that regulates Ab access to epitope F [25,32]. Additional factors, including inter-strain recombination [33] and polymerase fidelity [34,35], may also contribute to GII.4 predominance. Unfortunately, the lack of a well-established virus propagation method excludes evaluation of the effect of these factors on viral fitness. In contrast to the GII.4 strains, the other NoV genotypes do not appear to be evolving via epochal evolution.

The most significant obstacles to development of an effective NoV vaccine are the large number of antigenic variants, an incomplete understanding of the components of protective immunity, and the unknown effect of a lifetime of NoV pre-exposure history on vaccine response and, consequently, the unknown effect of vaccination on response to circulating NoVs. Further, the effect of host genetic susceptibility to NoV infection on vaccine performance is also undefined. A monovalent NoV GI.1 vaccine based on Norwalk virus VLPs was demonstrated to be well tolerated and effective at limiting the risk of Norwalk virus illness and infection upon oral challenge [23,36]. Two doses of this VLP candidate vaccine reduced the rate of symptomatic infection by 47% and the overall rate of infection by 26%. Serum blockade Ab titers above 200 were associated with a 72% reduction in frequency of illness and a 57% reduction in infection, providing evidence that pre-challenge blockade Ab titers correlated to protection following vaccination and challenge in human volunteers [23]. Despite these positive results, a threshold for protection using serum blockade Ab titer has not been established, and because the assay format is not yet standardized, it remains difficult to compare blockade titers from study to study.

These initial findings in monovalent VLP vaccination studies were expanded upon in a follow-up age and dose escalation study of a multivalent VLP candidate vaccine composed of GI.1 and a GII.4 consensus VLP (GII.4C). GII.4C is an engineered VLP composed of the consensus sequence of three NoV GII.4 strains that circulated in 2002 and 2006 (see Fig. 1). The multivalent vaccine was observed to be well tolerated and to elicit Ab responses in adults. Four dosages (5/5, 15/15, 50/50, and 150/150 μg GI.1/GII.4C VLP) of the multivalent vaccine were compared in a group of individuals aged 18–49 y, and the three highest dosages elicited similar Ab responses and reactogenicity [37]. Although secretor-negative participants had lower baseline titers against the vaccine antigens, they responded similarly to secretor-positive participants in titer and geometric mean fold rise (GMFR) [37], supporting in vitro [38] and in vivo [39–41] evidence that some GI and GII NoV strains bind to secretor-negative carbohydrates and infect the secretor-negative population. The 50/50-μg (GI.1/GII.4C) VLP dose was chosen for further study in a human challenge model with a GII.4.2002 strain [42]. Peak Ab titers to both vaccine components were observed after a single dose of vaccine, suggesting that vaccine-induced Ab responses may not rise above a maximum level. Despite high Ab responses, vaccinated individuals were not significantly protected from illness or infection compared to the placebo group. However, disease severity was significantly reduced. Interpretation of these results is complicated by a lower than expected infection rate and the absence of reporting of blockade Ab titers.

In this study we evaluated the potential cross-strain immunity induced by the multivalent candidate vaccine by measuring cross-reactive IgG and blockade Ab titers against the vaccine components, two additional GI VLPs, 2 GII VLPs, and five GII.4 VLPs not included in the vaccine formulation.

## Materials and Methods

### Ethics Statement

This follow-up study used de-identified human samples under University of North Carolina institutional review board exemption approval #11-0883. Original samples were collected by Takeda Vaccines under approval by institutional review boards at Saint Louis University School of Medicine, Saint Louis, Missouri; University of Rochester School of Medicine and Dentistry, Rochester, New York; and the Navy Medical Research Center, Silver Springs, Maryland, and participants provided written informed consent.

### Serum Samples

Serum samples were collected from participants in a NoV vaccine dose–response study conducted by Takeda Vaccines (Deerfield, Illinois) [37]. The results of that study guided selection of the 50-μg/VLP dose as the formulation to test in a human challenge trial (Table 1 and [42]). The 50-μg/VLP dose group of Cohort A (group A3) included ten adults aged 18–49 y vaccinated in January 2011 with an intramuscular injection of 50 μg of both GI.1 and GII.4C VLPs adjuvanted with 3-O-deacylated monophosphoryl lipid A (GlaxoSmithKline) and aluminum hydroxide (Brenntag Biosector, Denmark) on day 0 (dose 1), followed by an identical booster vaccination on day 28 (dose 2). All serum samples collected from the ten participants on day 0, 7, 21, 35, and 180 after dose 1 were evaluated. S1 and S2 Figs. list the total number of samples collected and evaluated for each VLP response by individual and assay. As a group, the Cohort A individuals receiving placebo (PBS without adjuvant) were sero-positive at baseline but did not mount serum responses to the vaccine antigens [37], therefore sera from this group were not included in further analyses of cross-reactive Ab responses. Serum samples were provided blinded of all participant identifiers. A set volume of each serum sample was provided for these exploratory studies.

**Table 1:** Study protocol for the original age and dose escalation study [37].

Cohort (Age Range)	Treatment Group and Dosage	Review Step	Schedule^a^						
			Day 0: Blood Draw and Dose 1	Day 7: Blood Draw	Day 21: Blood Draw	Day 28: Blood Draw and Dose 2	Day 35: Blood Draw	Day 56: Blood Draw	Day 180: Blood Draw
**Cohort A (18–49 y)**	Group A1; 5/5-μg VLP vaccine	Review of day 35 safety data from Group A1 then enrollment into Group A2	1,2,3,4,5	1,2,3	1	1,2,3,45	1,2,3	1,4,5	1,4,5
	Group A2; 15/15-μg VLP vaccine	Review of day 35 safety data from Group A2 then enrollment into Group A3	1,2,3,4,5	1,2,3	1	1,2,3,4,5	1,2,3	1,4,5	1,4,5
	Group A3; 50/50-μg VLP vaccine	Review of day 35 safety data from Group A3 then enrollment into Group A4	1,2,3,4,5	1,2,3	1	1,2,3,4,5	1,2,3	1,4,5	1,4,5
	Group A4; 150/150-μg VLP vaccine	Review of day 56 data from Cohort A then enrollment into Cohort B	1,2,3,4,5	1,2,3	1	1,2,3,4,5	1,2,3	1,4,5	1,4,5
	Placebo		1,2,3,4,5	1,2,3	1	1,2,3,4,5	1,2,3	1,4,5	1,4,5
**Cohort B (50–64 y)**	50/50-μg VLP vaccine	Review of day 35 data from Cohort B then enrollment into Cohort C	1	1	1	1	1	1	1
	Placebo		1	1	1	1	1	1	1
**Cohort C (65–85 y)**	50/50-μg VLP vaccine		1	1	1	1	1	1	1
	Placebo		1	1	1	1	1	1	1
**Cohort D (18–49 y)**	50/50-μg VLP vaccine	Simultaneous enrollment into Cohort D with enrollment in Cohort B and C	1	1	1	1	1	1	1
	Placebo		1	1	1	1	1	1	1

Group A3 was further evaluated in the current study.

^a^Numbers indicate data collected from blood draws: (1) serology for anti-NoV-specific GI.1 and GII.4 Ab titers, (2) Ab-secreting cells (ASCs) and homing markers (fresh peripheral blood mononuclear cells [PBMCs]—single site only), (3) ASCs (cryopreserved PBMCs—all sites in Cohort A), (4) memory B cells (cryopreserved PBMCs—all sites in Cohort A), and (5) cellular immune responses (cryopreserved PBMCs—all sites in Cohort A).

### Virus-Like Particles

**Fig 2:**
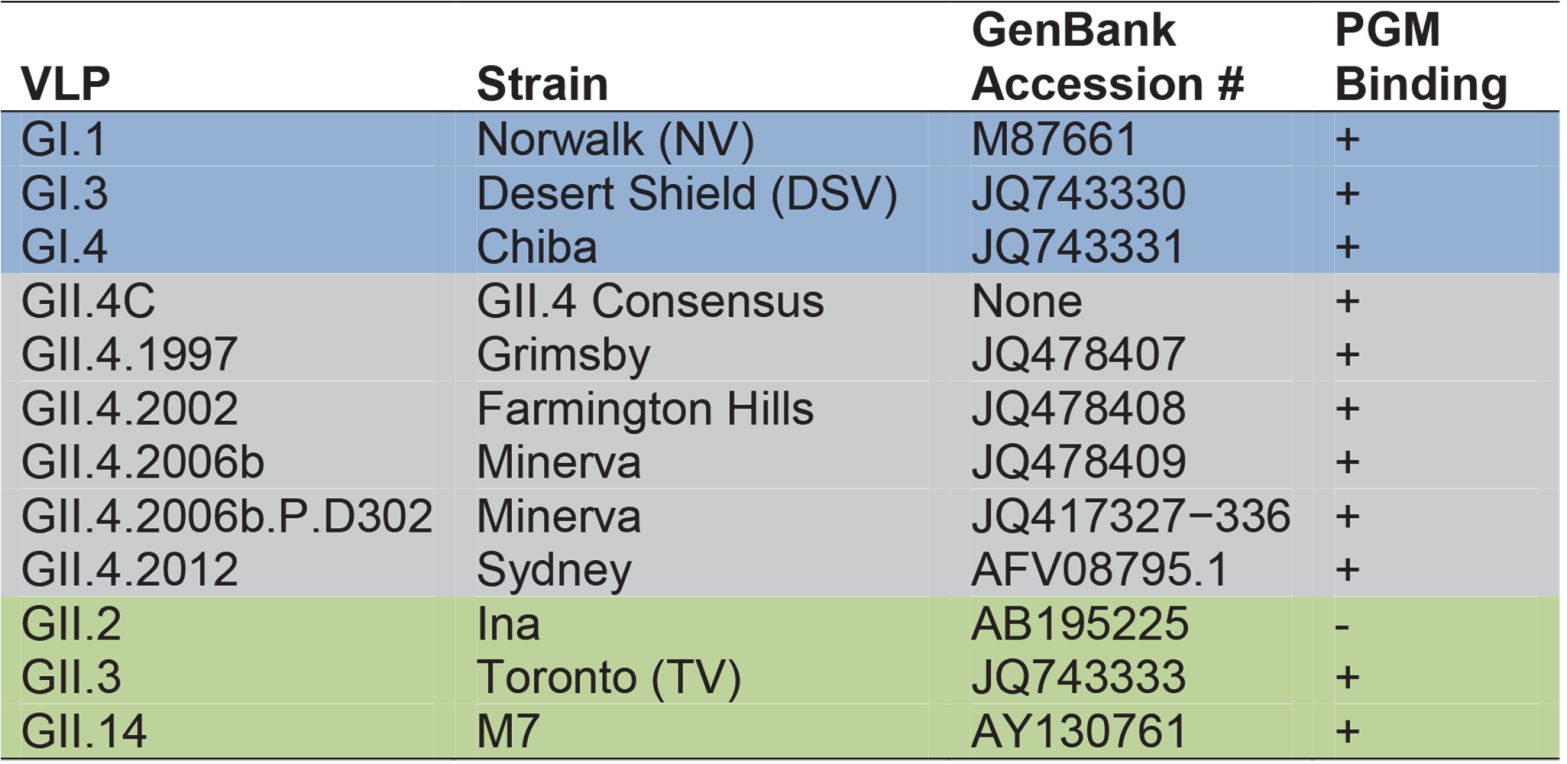
Characteristics of virus-like particles. Ab responses to a diverse panel of GI (blue), GII.4 (grey), and non-GII.4 GII (green) VLPs were compared in this study.

A diverse panel of VLPs was used in this study (Fig. 2). Except for GII.4C VLPs, ORF 2 genes were inserted directly into the VEE replicon vector for the production of virus replicon particles, as previously described [27,31]. VLPs were expressed in BHK cells and purified by velocity sedimentation in sucrose followed by simultaneous concentration and dialysis into PBS using 100-kDa MWCO centrifugal filter units (Millipore), if needed. GII.4C VLPs were provided by Takeda Vaccines and produced in the baculovirus system. VLP protein concentrations were determined by the BCA Protein Assay (Pierce). All of the VLPs used in this study bind to ligands found in porcine gastric mucin (PGM) type III, with the exception of GII.2. Although bioinformatics analyses do not suggest why, GII.2 does not bind to any identified HBGA sources except human type A and B saliva [43]; therefore, blockade assays were not performed for this VLP.

### Virus-Like-Particle-Specific Enzyme Immunoassay

EIA plates were coated with 0.25 μg/ml VLP in PBS before adding 2-fold serial dilutions of serum. The primary Ab incubation was followed by anti-human-IgG-HRP secondary Ab (GE Healthcare), and positive wells were color-developed with Ultra TMB substrate (Thermo Scientific). Plates were washed with PBS/0.05% Tween 20 (Thermo Scientific) between steps, and Ab dilutions were done in 5% Blotto/PBS/0.05% Tween 20. All incubations were done at room temperature. The percent maximum binding was calculated, sigmoidal dose–response curves fit to the data, and EC_50_ values determined using GraphPad Prism version 6.02 for Windows (GraphPad Software). All samples were tested in a minimum of two independent assays. EC_50_ titers below the lower limit of detection (100) were assigned a titer of 50.

### Carbohydrate-Binding Blockade Assay

As described previously [20,25], EIA plates were coated with 10 μg/ml PGM type III (Sigma Aldrich) diluted in PBS and blocked with 5% Blotto in PBS/0.05% Tween 20. VLPs (0.5 μg/ml) were pretreated with decreasing concentrations of serum for 1 h before being added to the carbohydrate-ligand-coated plates for 1 h. Ligand-bound VLP was detected with rabbit anti-GI or -GII or -GII.4 VLP hyperimmune serum followed by anti-rabbit-IgG-HRP (GE Healthcare). All tested VLPs, except for GII.4.2012 and GII.4.2006b.P.D302, were components of the VLP cocktails used to immunize rabbits. Multivalent GII.4 VLP immunization resulted in broadly reactive serum that recognizes GII.4.2012 and GII.4.2006b.P.D302 [44]. Wash steps, Ab dilutions, and color development were completed as described above. All incubations were done at room temperature. The percent control binding was defined as the binding level in the presence of Ab pretreatment divided by the binding level in the absence of Ab pretreatment multiplied by 100. Blockade data were fit using sigmoidal dose–response analysis of nonlinear data in GraphPad Prism 6.02. EC_50_ values were calculated for sera that demonstrated blockade of at least 50% at the dilution series (40–20480) tested. Sera that did not block 50% of binding at the highest concentration tested were assigned an EC_50_ of 20 for statistical comparison.

### Cartography

As described previously [45], we utilized multi-dimensional scaling (MDS) to analyze and visualize the relationships between Ab titers against VLPs across the serum samples from this study. This approach allows for data of large dimensions (e.g., multiple serological responses across a range of individuals) to be “shrunk” down to a more manageable number of dimensions for analysis, while maintaining accurate relationships between data points. Briefly, the IgG EC_50_ titers of each serum against a panel of VLPs (Fig. 2) were normalized to the maximum response of each serum, as well as to the maximum overall titers across sera (normalization method 1 [46]; the same normalization was used for the blockade Ab EC_50_ titers). We calculated Euclidean distances, *D*, between each pair of Ab titers against VLPs. We utilized MDS to then identify XYZ coordinates that maintain the underlying Euclidean distances. We used Matlab 8.1’s (MathWorks) cmdscale function for the MDS. We used R (http://www.r-project.org) and the rgl package for 3-D visualization of our data, as well as for statistical analysis of the antigenic cartography data. Because of the unique nature of the *D* values (that they belong to pairs of data points instead of to singular data points), in most cases we focus our statistical analyses on contrasting *D* values within specific groups of interest with *D* values between specific groups of interest (e.g., *D* values within either GI or GII VLPs versus *D* values between GI and GII VLPs). Unless noted otherwise, these tests take the form of a set of *t*-tests within a time point/assay combination, and we report as significant only those tests that pass an ad hoc Bonferroni correction (*p* = 0.05/number of tests done within a time point/assay combination).

### Blocking of Binding Assay

EIA plates were coated with 0.25 μg/ml GII.4.1997 or GII.4.2006b VLP, and serum samples were added to VLP-coated plates at serial 2-fold dilutions. After 1 h, mouse mAbs to epitope A (GII.4.1987.G1 [26] or GII.4.2006.G2 [26]) or epitope F (GII.4.2002.G5 [32]; MAB227P from Maine Biotechnology Services) were added and incubated at room temperature for 1 h. Bound mouse mAb was detected with sheep anti-mouse-IgG-HRP (GE Healthcare). Plates were washed, the color developed, and the amount of serum (1/serum dilution) needed to block 50% of the mAb binding (EC_50_) determined as described above for EIAs.

### Statistical Analysis

Statistical analyses were done using GraphPad Prism 6.02 and SAS (version 9.3) unless otherwise noted. For serum IgG and blockade Ab titers, geometric mean titer (GMT), GMFR from baseline, and the seroresponse frequency were calculated for each group. The changes in GMFR from baseline were tested using a paired *t*-test of the log values. Seroresponse was defined as a ≥4-fold increase above baseline titer. Generalized estimating equations (GEEs) were used to estimate the relative risk of (1) a 4-fold increase in blockade Ab titers at day 7 compared to day 0 given baseline titers above or below the assay limit of detection, for any VLP and (2) day 7 titers above or below the assay limit of detection given baseline titers above or below the assay limit of detection, for any VLP, along with corresponding chi-squared *p*-values. GEEs were used to account for the correlated data points (specimens collected at different follow-up time points from the same participant are correlated), as opposed to linear regression, which assumes independent data points.

## Results

### Vaccination with the Multivalent Virus-Like Particle Candidate Vaccine Results in Cross-Strain-Reactive IgG Responses

Based on results from a dose escalation study in adults aged 18–49 y (Table 1 and [37]), the 50-μg GI.1/GII.4C VLP vaccine formulation was chosen for further study. Complete demographic information for the group in the dose escalation study that received this dose (Group A3) compared to the other dose groups is provided in Table 2. As the vaccine target population will not be pre-screened for secretor status and some NoV strains infect secretor-negative individuals [39–41], secretor-negative participants were included in the larger dose escalation cohort and Group A3 (four out ten participants). Complete data comparing IgG and blockade Ab responses by secretor status, day, and VLP are provided in S1 and S2 Figs. As reported for the full study [37], Ab responses between the secretor-negative and secretor-positive participants in Group A3 were generally not significantly different in GMT or GMFR, beyond the exceptions discussed below.

**Table 2:** Demographics of the Cohort A dose groups in the dose escalation study.

Characteristic	Group A1: 5/5-μg VLP Vaccine (*n* = 10)	Group A2: 15/15-μg VLP Vaccine (*n* = 10)	Group A3: 50/50-μg VLP Vaccine (*n* = 10)	Group A4: 150/150-μg VLP Vaccine (*n* = 9)	Placebo (*n* = 9)	All Participants (*n* = 48)
**Gender**						
Male	3 (30)	4 (40)	3 (30)	2 (22)	1 (11)	13 (27)
Female	7 (70)	6 (60)	7 (70)	7 (78)	8 (89)	35 (73)
**Ethnicity**						
Non-Hispanic	8 (80)	10 (100)	10 (100)	9 (100)	9 (100)	46 (96)
Hispanic	2 (20)	0	0	0	0	2 (4)
**Race**						
American Indian/Alaskan Native	0	0	0	0	0	0
Asian	0	0	2 (20)	2 (22)	0	4 (8)
Hawaiian/Pacific Islander	0	0	0	0	0	0
Black/African American	2 (20)	2 (20)	2 (20)	1 (11)	2 (22)	9 (19)
White	8 (80)	7 (70)	6 (60)	6 (67)	7 (78)	34 (71)
Multi-racial	0	1 (10)	0	0	0	1 (2)
Other/unknown	0	0	0	0	0	0
**Age**						
Mean (standard deviation)	33 (9)	31 (11)	31 (8)	32 (9)	33 (13)	32 (10)
Median	32	30	37	34	27	32
Minimum, maximum	(23, 46)	(19, 46)	(22, 42)	(23, 49)	(19, 49)	(19, 49)
**Saliva secretor status**						
Positive	7 (70)	8 (80)	6 (60)	9 (100)	8 (89)	38 (79)
Negative	3 (30)	2 (20)	4 (40)	0	1 (11)	10 (21)
**Blood type**						
A	3 (30)	3 (30)	5 (50)	3 (33)	2 (22)	16 (33)
B	0	1 (10)	0	1 (11)	1 (11)	3 (6)
O	6 (60)	4 (40)	5 (50)	5 (56)	5 (56)	25 (52)
AB	1 (10)	2 (20)	0	0	1 (11)	4 (8)

Data are given as *n* (percent), except for age data. Group A3 was studied in this manuscript.

To measure the potential cross-strain protection provided by the multivalent NoV VLP vaccine, we retrospectively analyzed serum samples from the ten participants in the 50-μg dose group from the dose escalation study for cross-reactive IgG and blockade Ab titer to VLPs representing three different GI genotypes; five time-ordered, antigenically distinct GII.4 strains; and two additional non-GII.4 GII genotypes (Fig. 2). Participants received a dose of adjuvanted vaccine on day 0 and day 28. Sera collected on day 0 (before vaccine dose 1), day 7 after vaccine dose 1, day 35 (7 d after vaccine dose 2), and day 180 (6 mo after vaccine dose 1) were evaluated from each of the ten participants. One participant failed to return for the day 180 sample collection.

**Fig 3:**
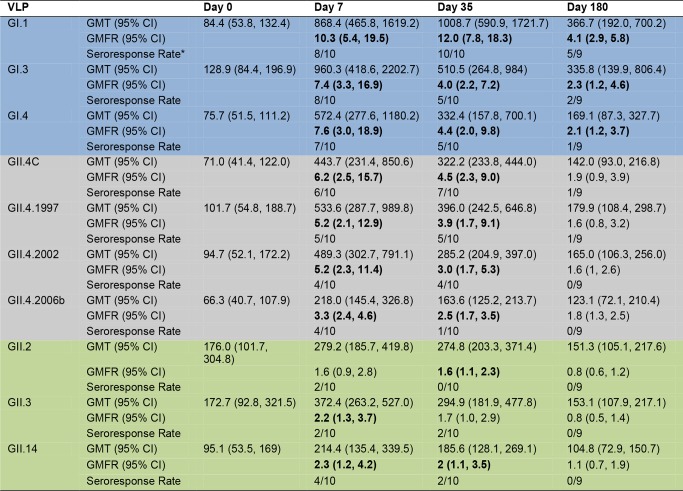
Mean EC_50_ IgG titer in vaccinated participants. Serum samples collected from participants who received the 50/50-μg VLP dose were assayed for IgG reactivity to a panel of GI (blue), GII.4 (grey), and non-GII.4 GII (green) VLPs. The seroresponse rate is the ratio of the number of participants with a ≥4-fold titer increase above day 0 titer compared to the total number of samples tested at day 0 for each VLP. Bolded values denote significant increases in GMFR above baseline.

As a group (Fig. 3), the baseline IgG GMTs to GI types were relatively low, with significant increases in GMFRs at day 7, 35, and 180 after dose 1. Titers peaked at day 7 post-vaccination, rising 10.3-fold for GI.1, 7.4-fold for GI.3, and 7.6-fold for GI.4. Despite a booster vaccination at day 28, day 35 IgG titers were generally lower than day 7 titers, with GMFRs of 12-, 4-. and 4.4-fold for GI.1, GI.3, and GI.4, respectively. At day 180, GMFRs were 4.1-fold for GI.1, 2.3-fold for GI.3, and 2.1-fold for GI.4. Secretor-negative participants had significantly less titer to GI.4 at day 0 and to GI.1 at day 0 and day 180, possibly reflecting less infection history with these strains (S1 Fig.). At day 7, the IgG seroresponse rate was 8/10 for GI.1 and GI.3 and 7/10 for GI.4 (Figs. 3 and S3). At day 35, 10/10 participants seroresponded for GI.1, and 5/10 for the other GI VLPs, and these responses persisted at day 180 in 5/9 participants for GI.1, 2/9 for GI.3, and 1/9 for GI.4.

The IgG response among different strains of GII.4 VLPs was relatively consistent (Fig. 3), with no significant differences in GMFR between GII.4 VLPs at any time point. As a group, GII.4 baseline GMTs were relatively low and in the same range as the GI titers, with significant increases in GMFR at day 7 and day 35 after dose 1 for all GII.4 VLPs. Titers generally peaked at day 7 post-vaccination, rising 6.2-fold for GII.4C, 5.2-fold for GII.4.1997, 5.2-fold for GII.4.2002, and 3.3-fold for GII.4.2006b. As demonstrated for GI VLPs, despite a booster vaccination at day 28, day 35 titers were lower than day 7 titers, with GMFRs of 4.5 for GII.4C, 3.9 for GII.4.1997, 3.0 for 2002, and 2.5 for GII.4.2006b. At day 180, IgG titers were less than 2-fold above baseline for all of the GII.4 VLPs. Secretor-negative and -positive participants responded similarly to GII.4 VLPs (S1 Fig.). As individuals (S3 Fig.), at day 7, 6/10 participants seroresponded to GII.4C, 5/10 to GII.4.1997, and 4/10 to GII.4.2002 and GII.4.2006b VLPs. At day 35, the response rates were 7/10, 5/10, 4/10, and 1/10, respectively. At day 180, 1/9 participants maintained a seroresponse to GII.4C and GII.4.1997.

Increases in cross-reactive IgG were detected to GII.2, GII.3, and GII.14 VLPs after vaccination. As a group (Fig. 3), a ≥4-fold increase in titer was not detected for any of the non-GII.4 GII VLPs at any time point. Some individuals did serorespond to each of the non-GII.4 GII VLPs, although none of the responses persisted to day 180 (S3 Fig.). GMTs were not significantly different for any non-GII.4 GII VLPs at any time point. Despite this observation, secretor status significantly impacted the GMFRs to non-GII.4 GII VLPs. Secretor-negative participants had significantly higher GMFRs on day 7 and 35 to GII.2, GII.3, and GII.14 (S1 Fig.). Together, these data indicate that the multivalent vaccine induces production of IgG that reacts with not only the immunizing strain VLPs but also VLPs from strains and genotypes not included in the vaccine. Maximum serum IgG levels were seen after a single dose of vaccine, and serum IgG was not equally reactive with all NoV genotypes.

### Vaccination with the Multivalent Virus-Like Particle Candidate Vaccine Results in Cross-Strain Blockade Antibody Responses

**Fig 4:**
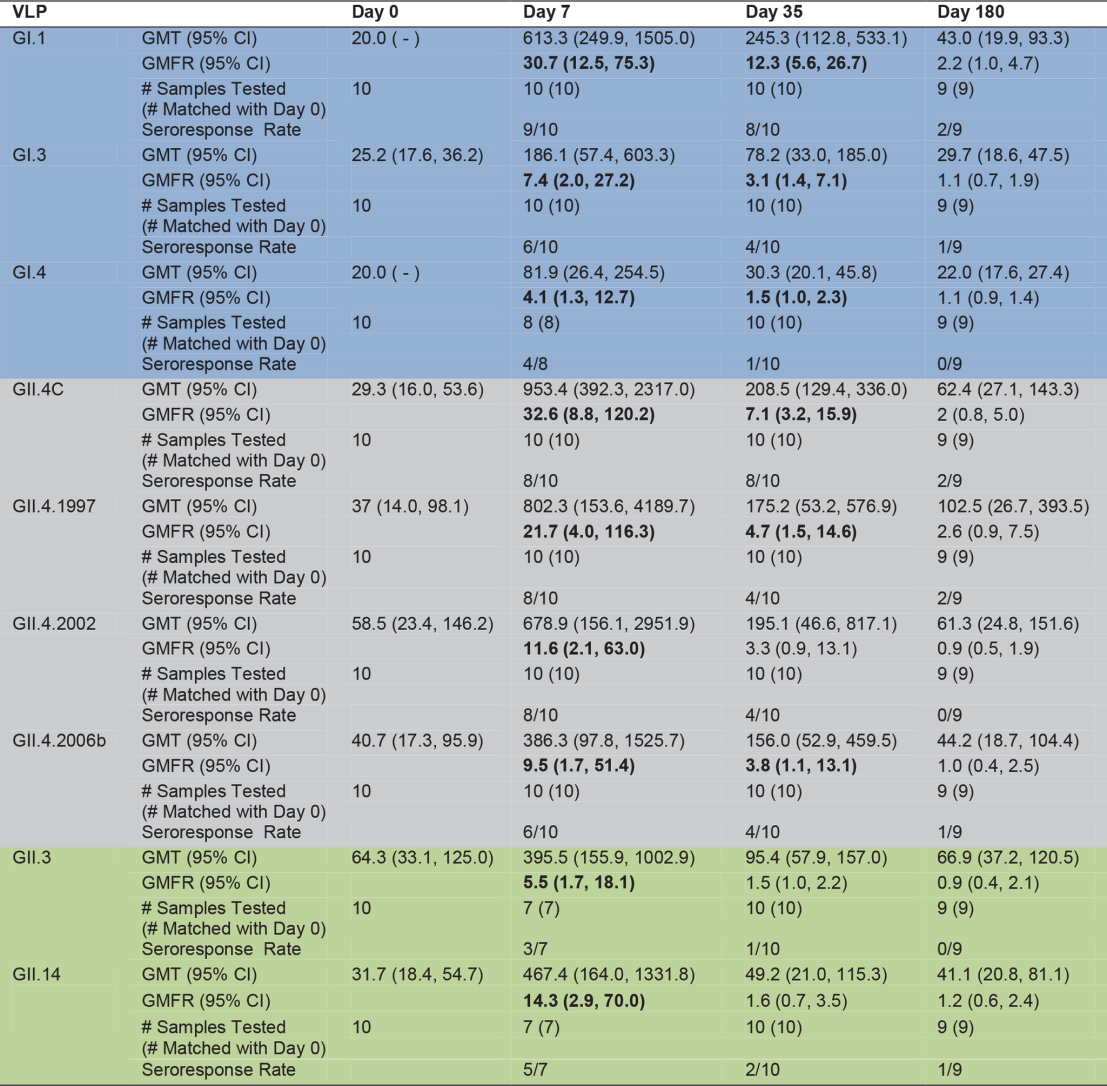
Mean EC_50_ blockade antibody titer in vaccinated participants. Serum samples collected from participants who received the 50/50-μg VLP dose were assayed for blockade Ab to a panel of GI (blue), GII.4 (grey), and non-GII.4 GII (green) VLPs. The seroresponse rate is the ratio of the number of participants with a ≥4-fold titer increase above day 0 titer compared to the total number of samples tested at day 0 for each VLP. Bolded values denote significant increases in GMFR above baseline.

The inherent cross-strain reactivity of serum IgG demonstrated above and elsewhere [19,41] has confounded interpretation of the association between IgG and risk of NoV infection. However, the specificity of measuring blockade Abs demonstrated a clear correlation between titer and protection both from GI.1 symptomatic infection and reinfection with homotypic virus in challenge studies [23]. Therefore, we tested the serum samples for blockade Ab titers against the panel of GI, GII.4, and non-GII.4 GII VLPs as a predictor of cross-strain protection after vaccination. As a group, there were no significant differences in blockade Ab titer between any VLP pair at day 0. Day 0 geometric mean blockade Ab titers to GI VLPs were low, with significant increases in GMFRs at day 7 and 35 for all three GI VLPS (Fig. 4). Titers peaked at day 7 post-vaccination, rising 30.7-fold above baseline for GI.1, 7.4-fold for GI.3, and 4.1-fold for GI.4. At day 35, GMFRs were 12.3 for GI.1, 3.1 for GI.3, and 1.5 for GI.4. Blockade Ab titers returned to baseline levels at day 180. GI blockade Ab GMFRs were not dependent on secretor status (S2 Fig.). Individually (S4 Fig.), at day 7, 9/10 participants responded with a ≥4-fold increase in blockade Ab titer to GI.1, 6/10 to GI.3, and 4/8 to GI.4. At day 35, elevated titers were maintained in 8/10 participants to GI.1, 4/10 to GI.3, and 1/10 to GI.4. At day 180, 2/9 participants maintained a ≥4-fold increase in blockade Ab titer to GI.1, and 1/9 to GI.3.

The nearly 100% NoV seropositivity in adults indicates wide NoV exposure across the population [38]. Because Ab matures through somatic hypermutation (SHM) after each additional antigen encounter [47], we evaluated the ratio of blockade Ab to reactive IgG for each VLP as a potential marker of the specificity and maturity of the vaccine-induced Ab response amongst the panel of VLPs. A ratio ≥ 1 suggests that serum may have more blockade activity per IgG activity and thus may be more mature and the result of a repeat exposure. The ratio of blockade Ab to IgG increased ∼3-fold above baseline for GI.1 at day 7, while remaining unchanged for GI.3 and GI.4 (S5 Fig.). These data suggest that vaccination may primarily induce production of Abs with enhanced potency for GI.1 blockade, likely because of SHM of preexisting cross-GI blockade Abs that were affinity-matured against GI.1 post-vaccine exposure, while maintaining some fraction of cross-strain blockade Abs.

For GII.4 VLPs (Fig. 4), the day 0 geometric mean blockade Ab titers were relatively low, with significant increases in GMFRs at day 7 for all GII.4 VLPs and at day 35 for GII.4C, GII.4.1997, and GII.4.2006b. Blockade titers were similar across the panel of different GII.4 strains, with no significant differences in GMFR at any time point. Titers peaked at day 7 post-vaccination, rising 32.6-fold for GII.4C, 21.7-fold for GII.4.1997, 11.6-fold for GII.4.2002, and 9.5-fold for GII.4.2006b. Day 35 GMFRs for blockade Ab titers were 7.1 for GII.4C, 4.7 for GII.4.1997, 3.3 for GII.4.2002, and 3.8 for GII.4.2006b. At day 180, blockade Ab titers approached baseline (1.1- to 2.6-fold) for all of the GII.4 VLPs. Secretor-negative participants responded similarly to secretor-positive participants across the GII.4 panel (S2 Fig.). Individually (S4 Fig.), 8/10 participants responded with a ≥4-fold increase in blockade Ab titer to GII.4C at day 7 and day 35. At day 180, ≥4-fold titers were maintained in 2/9 of the participants. Similar high levels of fold increase above baseline blockade Ab titer extended across the GII.4 panel at day 7 (GII.4.1997 and GII.4.2002, 8/10; GII.4.2006b, 6/10). By day 35, 4/10 of participants maintained titers ≥4-fold above baseline for GII.4.1997, GII.4.2002, and GII.4.2006b. At day 180, 2/9 participants sustained ≥4-fold titers to GII.4.1997, and 1/9 participants to GII.4.2006b. Notably, the ratio of blockade Ab to IgG at day 7 compared to the baseline ratio increased for all GII.4 VLPs (fold increase: GII.4C, 5.2; GII.4.1997, 4.1; GII.4.2002, 2.2; GII.4.2006b, 2.9) (S5 Fig.), suggesting that vaccination induces a broad GII.4-reactive response despite strain evolution over the 15-y period represented in the panel.

Baseline blockade Ab titers to GII.3 and GII.14 non-vaccine types were low and had significant increases in GMFR only on day 7. As a group (Fig. 4), titers peaked at day 7 at 5.5-fold (GII.3) and 14.3-fold (GII.14) above baseline. Elevated blockade Ab titers were not boosted or maintained at day 35 or 180, although some individuals did maintain ≥4-fold increases at these later time points (S4 Fig.). Contrary to IgG findings, secretor-negative status did not associate with higher GMFR in blockade Ab titer post-vaccination (S2 Fig.). The ratio of blockade Ab to IgG increased for both VLPs at day 7 compared to day 0 (fold increase: GII.3, 3.1; GII.14, 6.8) (S5 Fig.) before returning to baseline ratios, supporting GII.4 VLP data indicating that vaccination induces a rapid, broad GII Ab response.

### Elevated Preexisting Blockade Antibody Titers against Any Norovirus Virus-Like Particles Do Not Prevent a Vaccine Response

**Fig 5:**
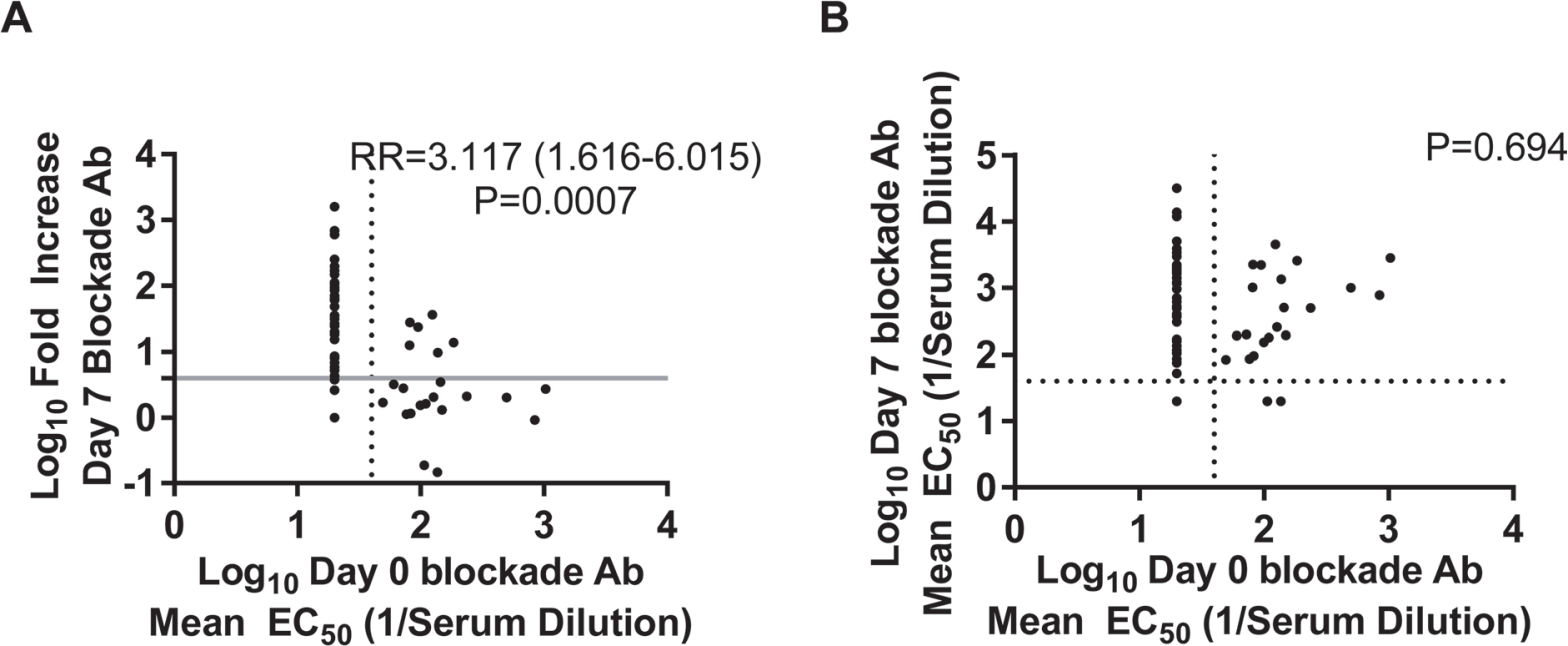
Day 0 blockade antibody titers below the assay limit of detection for any norovirus virus-like particle are predictive of a ≥4-fold increase, but not overall blockade Ab titer, at day 7. Day 0 blockade Ab titers for all of the NoV VLPs studied were compared to the corresponding day 7 fold increase (A) and titer of blockade Ab (B) using GEEs (*n* = 10 participants, 82 samples). The likelihood of responding to vaccination with a ≥4-fold increase in blockade Ab titer to any NoV VLP was 3-fold greater if the day 0 titer was below the assay limit of detection. Day 0 blockade Ab titer did not correspond with day 7 titer. The dotted lines mark the lower limit of detection of the blockade Ab assay. The solid grey line marks a 4-fold increase in blockade titer at day 7. RR, relative risk.

Preexisting blockade Ab titers have been shown to correlate with protection from GI.1 infection and illness in a NoV challenge study [23]; however, the effect of preexisting Ab on vaccine response is unknown. The absence of a titer boost after the second vaccine dose suggests that preexisting Ab titers may have a limiting effect on the magnitude of subsequent vaccine responses or that vaccine-induced Ab titers plateau [37,42]. To determine whether elevated blockade Ab titer in the 50-μg dose group may have impacted the multivalent VLP vaccine response, we evaluated the association between day 0 blockade Ab titers to any of the NoV VLPs and the likelihood of developing a ≥4-fold increase in blockade Ab titer. The lack of preexisting blockade Ab to any NoV VLP tested significantly correlated with increased vaccine response by day 7 (*p* < 0.001, chi-squared test) (Fig. 5A). Independent of VLP tested, 51 out of 57 (89.5%) samples with a ≥4-fold increase in blockade Ab titer post-vaccination had no detectable VLP-specific blockade Ab titer at day 0. Sera with baseline blockade Ab titer below the limit of detection were 2.6 (excluding vaccine VLP responses for the analysis) or 3.1 times (including all VLPs) more likely to have a ≥4-fold increase in blockade Ab titer to the same VLP at day 7 post-vaccination compared to participants with higher blockade Ab titers. However, further analyses of the relationship between day 0 blockade Ab titers and day 7 titers revealed no significant association (Fig. 5B), regardless of inclusion of vaccine VLP responses, as participants with higher baseline titers also responded with an increase in titer of ≥4-fold at day 7. Together these data show that the presence of preexisting blockade Ab does not prevent a NoV blockade Ab response to vaccination, but instead that an Ab titer plateau can be reached regardless of baseline titer.

### Vaccination with the Multivalent Virus-Like Particle Candidate Vaccine Induces Antibody That Blocks Novel GII.4 Norovirus Strain Virus-Like Particles

**Fig 6:**
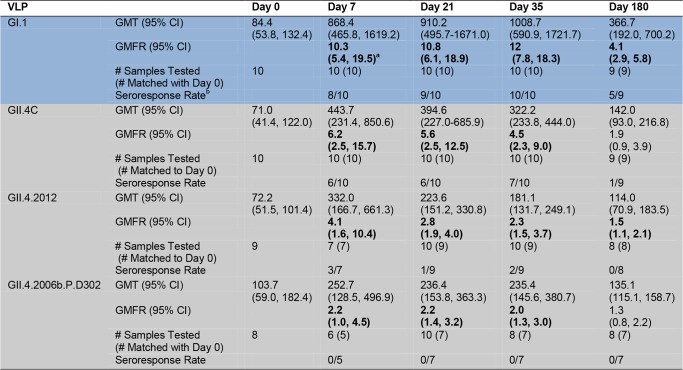
Mean EC_50_ IgG titers to novel GII.4 strain virus-like particles. Serum samples collected from participants who received the 50/50-μg VLP dose were assayed for IgG reactivity to the vaccine components and to two novel GII.4 VLPs. GI VLP is shaded blue; GII.4 VLPs are shaded grey. The seroresponse rate is the ratio of the number of participants with a ≥4-fold titer increase above day 0 titer compared to the total number of samples tested at day 0 for each VLP. Bolded values denote significant increases in GMFR above baseline.

**Fig 7:**
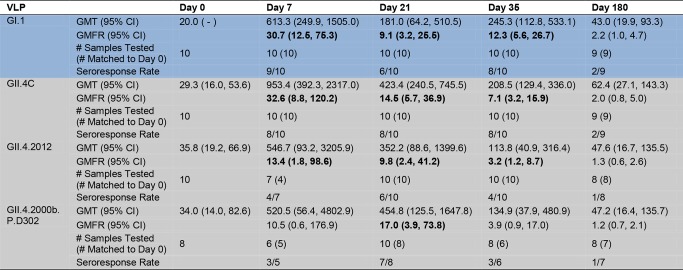
Mean EC_50_ blockade antibody titers to novel GII.4 strain virus-like particles. Serum samples collected from participants who received the 50/50-μg VLP dose were assayed for blockade Ab to the vaccine components and to two novel GII.4 VLPs. GI VLP is shaded blue; GII.4 VLPs are shaded grey. The seroresponse rate is the ratio of the number of participants with a ≥4-fold titer increase above day 0 titer compared to the total number of samples tested at day 0 for each VLP. Bolded values denote significant increases in GMFR above baseline.

**Fig 8:**
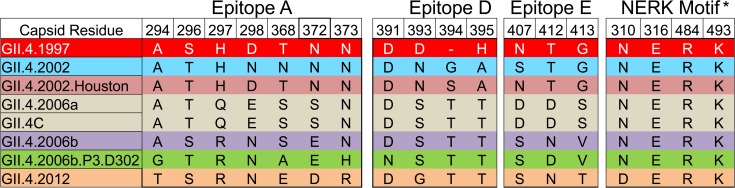
Amino acid sequence of identified GII.4 blockade antibody epitopes (A, D, and E) and the regulating domain of epitope F (NERK motif) in GII.4 virus-like particles relevant to this study. Color indicates antigenic groupings based on epitope A sequence. *The amino acid coordinates of epitope F are unknown. The NERK motif is a temperature-sensitive regulator of Ab access to epitope F [32].

The data described above clearly indicate that vaccination induces a boost in blockade Ab across GII.4 VLPs that circulated before the vaccination program. To evaluate Ab activity against a newly emergent GII.4 strain for which preexisting immunity is unlikely, we tested the serum samples for reactive IgG and blockade Ab titer against two novel GII.4 strain VLPs (Figs. 1 and 6–8). GII.4.2012 began circulating globally in 2012, more than a year after the trial sample collection was completed. GII.4.2006b.P.D302 VLP represents a unique GII.4 strain sequenced from an immune-compromised patient with symptomatic NoV infection for over 1 y [45,48]. Importantly, GII.4.2006b.P.D302 is divergent from GII.4.2006b at all three evolving blockade epitopes, resulting in loss of Ab blocking activity of mouse and human polyclonal sera and mAbs (Fig. 8 and [45]). As the extensive cross-reactivity studies described above had depleted the finite volume of each serum sample that had been set aside for these exploratory studies, we included day 21 serum samples from the same participants in the novel GII.4 VLP studies. Consequently, responses to the novel GII.4 VLPs are likely impacted by sample number loss due to volume depletion (Figs. 6, 7, and S1–S4). Similar to the titers for the other GII.4 strains, baseline reactive IgG GMTs to GII.4.2012 and GII.4.2006b.P.D302 were relatively low, with significant increases in GMFRs at day 7, 21, 35, and 180 to GII.4.2012 (Fig. 6). GII.4.2012 titers peaked at 4.1-fold on day 7, followed by 2.8-fold at day 21, 2.3-fold at day 35, and 1.5-fold at day 180 after dose 1. IgG GMFRs to both novel GII.4 VLPs were similar between secretor-negative and -positive participants (S1 Fig.) Individually, GII.4.2012 seroresponse rates were 3/7 at day 7, 1/9 at day 21, 2/9 at day 35, and 0/7 at day 180 (S3 Fig.). IgG GMFRs to GII.4.2006b.P.D302 were significant on day 7, 21, and 35, rising 2.2-, 2.2-, and 2.0-fold, respectively. Unlike for the other VLPs tested, GII.4.2006b.P.D302 IgG titers did not change ≥4-fold with vaccination at any time point (Figs. 6 and S3).

Similar to IgG titers, as a group, day 0 geometric mean blockade Ab titers against both novel GII.4 VLPs were low, with significant increases in GMFR on day 7, 21, and 35 for GII.4.2012 (Fig. 7). GII.4.2012 titers peaked at day 7 post-vaccination, rising 13.4-fold, followed by 9.8-fold on day 21 and 3.2-fold on day 35, before returning to baseline on day 180. Blockade Ab GMFRs to both novel GII.4 VLPs were similar between secretor-negative and -positive participants (S2 Fig.). Individually (S4 Fig.), 4/7 participants had a ≥4-fold increase in blockade Ab titer to GII.4.2012 at day 7, 6/10 at day 21, 4/10 at day 35, and 1/8 at day 180. GII.4.2006b.P.D302 titers peaked at day 21 at 17.0-fold above baseline. Day 35 titers were 3.9-fold above baseline. The lack of significant response at day 7 and day 35 is likely impacted by additional sample depletion (Figs. 7, S2, and S4). At day 180, blockade Ab titers had returned to near baseline. Individually (S4 Fig.), 3/5 participants had a ≥4-fold increase in blockade Ab titer to GII.4.2006b.P.D302 at day 7, 7/8 at day 21, 3/6 at day 35, and 1/7 at day 180. The ratio of blockade Ab to IgG increased for both VLPs at day 7 compared to day 0 (S5 Fig.) before returning to baseline ratios. As identified for the other GII.4 VLPs, the ratio of blockade Ab to IgG was greater than one through day 35, indicating that the GII.4.2012 and GII.4.2006b.P.D302 blockade Ab may be mature Ab derived from previous NoV exposures. Importantly, these data demonstrate that NoV multivalent VLP vaccination can induce a blockade Ab response to a GII.4 strain unknown at the time of vaccination, suggesting that broad-based NoV immunity by vaccination is an achievable goal.

### Multivalent Virus-Like Particle Vaccine-Induced Antibody Responses Associate with Antibody Responses to Early (1997–2002) GII.4 Strains

**Fig 9:**
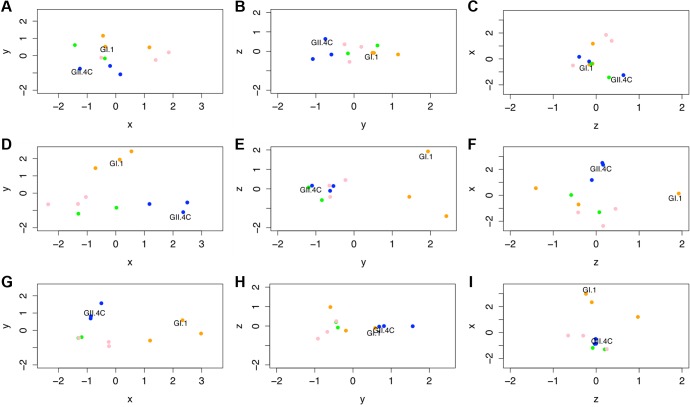
Evolving IgG responses to virus-like particles throughout the time course of the study. Individual points represent VLPs, and the distances between points represent the overall differences in the magnitude of IgG responses from all ten participants against these NoV VLPs on day 0 (A–C), day 7 (D–F), and day 180 (G–I). Specifically, the distance between VLPs (with each unit in any dimension [*D*] relating to a 3.4-fold difference in total IgG responses) shows how similar total IgG responses across the ten participants were to each pair of VLPs tested within this study and how vaccine components (noted in each panel) cluster with other NoV strain VLPs. Despite similarities across these responses, we are able to show that GI VLPs (orange) cluster with the GI.1 vaccine component, early GII.4 VLPs (blue) cluster with the GII.4C vaccine component, and late GII.4 VLPs (green) and the other GII VLPs (pink) cluster together and away from the other VLPs. For each time point, the *x*-axis is that showing the most variation between all VLPs, then the *y*-axis, then the *z*-axis. Therefore, down each column, we can see how immune responses change and track through the time course of the study. Of note are the clusterings of each virus subtype through these responses.

To test for any relationship between the vaccine components and other NoV VLPs that may be indicative of shared epitopes, we first compared the antigenic relationship of the panel of NoV VLPs based on IgG reactivity and blockade Ab titer using MDS computational and statistical approaches. These approaches are a powerful tool to help visualize and analyze similarities between samples across a range of variables, in this case, analyzing the similarities between different VLPs (samples) based on their likelihood of an immune response being raised across a range of individuals (variables). In order to assess the overall similarities that pairs of VLPs exhibited across sera from vaccinated individuals, we calculated Euclidean distances, *D* (see S1 Table), between each VLP pair at each time point. Euclidean distances are calculated such that two VLPs possessing identical EC_50_ values for each tested serum will have a *D* of 0; with increasing dissimilarity in the responses these VLPs share within any specific sera, *D* will increase. In our analysis of IgG similarities, each *D* unit equaled roughly a 3.4-fold difference in overall serum response. We utilized MDS to identify XYZ coordinates for each VLP that accurately capture the *D* values between VLPs, as described previously [46,49]. This approach allowed us to visualize and analyze the cross-reactivity of the IgG response over the time course of this study (Fig. 9).

At day 0 before vaccination, the NoV VLPs were generally clustered together and did not sort by subtypes (GI versus early GII.4 [1997 and 2002] versus late GII.4 [2006b and 2012] versus other GII genotype VLPs; Fig. 9A–C), with the average *D* between VLPs being 1.79 (range 0.55–3.34). At day 7, the peak of vaccine response (Fig. 9D–F), the distance between VLPs expanded, with an average *D* of 3.07 (range 1.12–4.89), illustrating the fact that individual vaccine recipients mounted responses to aspects of the vaccine, but that these responses were not universal across VLPs (if this were the case, all VLPs would still remain closely clustered), specifically when examining VLPs not included in the vaccine formulation. As could be seen when examining the primary IgG response to GI.1, there was a distinct response to this vaccine component. Utilization of antigenic cartography allowed us to show that the responses to this VLP were quite distinct from those raised against other VLPs. In particular, the *D* values between GI.1 and all GII viruses (average *D* = 3.79, range 3.0–4.24) show that the response raised against the GI.1 component of the vaccine was fairly independent of the responses raised against GII VLPs. While not attaining multiple-test-correcting significance (*t*_25_ = 4.795, *p* = 0.038), the effect of cross-reactivity between GI.1 and the other GI VLPs was apparent in our analysis. These VLPs are individually fairly distant from GI.1 (*D* of 3.4 and 2.7), but as a trio they segregate away from the GII VLPs (average *D* = 3.59, range 2.24–4.56). This result illustrates our ability to detect even weak secondary responses against these dissimilar GI VLPs by utilizing antigenic cartographic approaches.

As described above, GII VLPs were distinct from the GI VLPs at 7 d post-vaccination. Interestingly, the GII.4C VLP (representing the vaccine component) associated much more closely with the early GII.4 VLPs than with either the late GII.4 VLPs or the GII VLPs from other genotypes (ANOVA: *F*_2,4_ = 14.74, *p* = 0.0143), showing that the IgG response to GII.4C vaccination is highly cross-reactive with the early GII.4 VLPs but not the antigenically distinct contemporary GII.4 VLPs or other GII VLPs. Even as late as 180 d post-vaccination (Fig. 9G–I), clustering by genogroup (or by subclades within genogroups) remained (*t*-test: *t*_53_ = 23.96, *p* < 0.001), although overall distances (dissimilarities) in IgG responses differentiating between VLPs had decreased compared to day 7. Specifically, the GI viruses clustered relative to the other virus strains (*t*_25_ = 11.32, *p* = 0.0024), GII.4C continued to cluster with GII.4.1997 and GII.4.2002 (*t*_25_ = 8.973, *p* = 0.0061), and the contemporary GII.4 VLPs remained tightly clustered with the other GII genotypes (*t*_38_ = 19.24, *p* < 0.001). Interestingly, and echoing our findings above, GII.4.1997 remained somewhat distinct from GII.4C and GII.4.2002 (Fig. 9G–I) because of the elevated levels of IgG against GII.4.1997 VLP still found in several of the vaccine recipients 6 mo post-vaccination.

**Fig 10:**
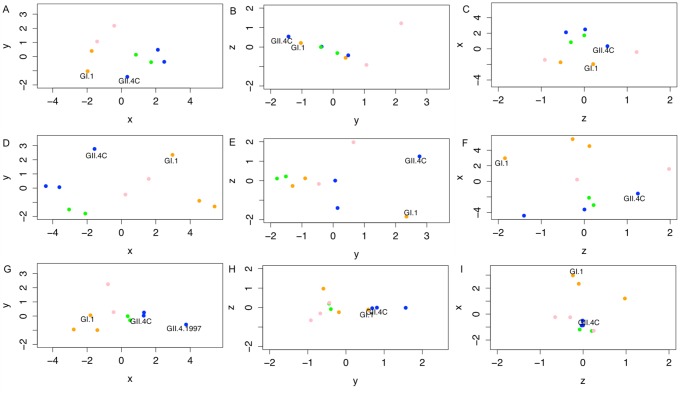
Evolving blockade antibody responses to virus-like particles throughout the time course of the study. Individual points represent VLPs, and the distances between points represent the overall differences in the magnitude of blockade Ab responses from all ten participants against these NoV VLPs on day 0 (A–C), day 7 (D–F), and day 180 (G–I). Specifically, the distance between VLPs (with each unit in any dimension [*D*] relating to a 3.2-fold difference in blockade Ab, or EC_50_ response) shows how similar total blockade Ab responses across the ten participants were between each pair of VLPs tested within this study, and how vaccine components (noted in each panel) cluster with other NoV strain VLPs. Across these responses, we are able to show that GI VLPs (orange) cluster with the GI.1 vaccine component, early GII.4 VLPs (blue) cluster with the GII.4C vaccine component, and the late GII.4 VLPs (green) and the other GII VLPs (pink) cluster together and away from the other VLPs. For each time point, the *x*-axis is that showing the most variation between all VLPs, then the *y*-axis, then the *z*-axis. Therefore, down each column, we can see how immune responses change and track through the time course of the study. Of note are the clusterings of each virus subtype through time, although with clear distinction of the vaccine components at day 7. At day 180, as titers have fallen, the blockade Ab distinctions between VLPs are diminished across the panels (G–I), with the exception of responses to GII.4.1997, suggesting that a memory Ab response to this strain may be driving the GII.4-reactive vaccine response.

Similar analyses using blockade Ab titers rather than IgG titers supported several of these findings, with the *D* values calculated for blockade titers corresponding to roughly a 3.2-fold change in blockade levels. In contrast to the tight clustering of VLPs in the EIA-reactive IgG before vaccination, the spread of the distances between VLPs was greater (average *D* = 2.88, range 0–4.52), indicative of the strain specificity of blockade Ab as well as the individual-to-individual variation in pre-immunization blockade levels (Fig. 10). There was some limited cross-reactivity even at day 0 (Fig. 10A–C), with subtypes being significantly more related than genotypes (*t*_43_ = 11.99, *p* = 0.001; within-subtype average *D* = 1.77, range 0–2.79; between-subtype average *D* = 3.13, range 0.77–4.52).

At 7 d post-vaccination (Fig. 10D–F), there was a wider range of *D* values (average *D* = 5.43, range 1.86–10.10). There was still a significant relationship between subtypes (*t*_43_ = 9.327, *p* = 0.0039), although the strong participant-specific responses to some VLPs obscured some of these groupings. Genotype grouping could not be definitively assigned to any given vaccine responses, as the GI VLPs remained only moderately clustered together (*t*_22_ = 7.517, *p* = 0.011; multiple-test cutoff is *p* = 0.0166), and while the GII VLPs clustered together (*t*_40_ = 40.11, *p* < 0.001), the GII.4C VLP did not cluster with any given GII subgroup. Consistent with our other observations above, while we cannot assign statistical confidence to this observation (as there is only a single *D* value to contrast), the two vaccine components (GI.1 and GII.4C) appeared to slightly co-segregate within the three-dimensional space (Fig. 10D–F), corresponding to the strong vaccine responses driven across vaccine recipients.

At day 180 (Fig. 10G–I), the distance between VLP clusters decreased (average *D* = 3.07, range 1.19–6.57), but was still elevated relative to pre-vaccine levels. In contrast to other time points, there was only a very moderate subgroup-based clustering (*t*_43_ = 4.277, *p* = 0.045), which could not be attributed to any given subgroup. Most of the variation within these responses is due to the maintenance of blockade Ab to GII.4.1997 VLP (*t*_43_ = 29.8, *p* < 0.001). These data support our premise that the GI.1 response can be characterized as an immature Ab response while the GII.4C response can be characterized as a memory Ab response driven by Abs that react primarily with epitopes present in GII.4.1997. We arrive at this conclusion specifically because blockade Ab responses are much stronger for GII.4.1997 than even for the vaccine component GII.4C.

### Vaccination with the Multivalent Virus-Like Particle Candidate Vaccine Induces Blockade Antibody Responses to Multiple Epitopes Cross-Reactive between Antigenically Divergent GII.4 Virus-Like Particles

**Fig 11:**
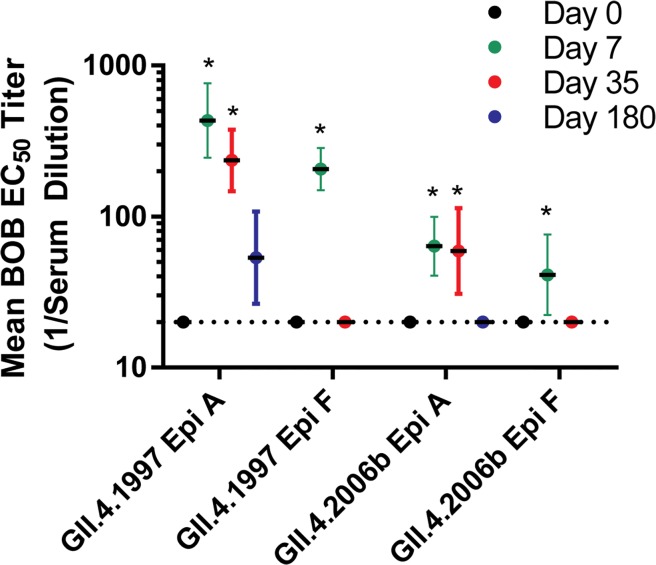
Vaccination results in a rapid but transient increase in antibody titer to multiple blockade epitopes in multiple GII.4 strains. Serum samples were evaluated for ability to block binding of mouse mAbs to epitope (Epi) A or F in GII.4.1997 and GII.4.2006b using a BOB assay. Sigmoidal curves were fit to the mean percent control binding (percent of mouse mAb bound to VLP in the presence of serum pretreatment compared to the amount of mouse mAb bound in the absence of serum pretreatment), and the mean EC_50_ titer (1/serum dilution) for BOB calculated. Dotted line marks 0.5 times the assay limit of detection. Error bars represent 95% confidence intervals. An asterisk indicates that the EC_50_ titer is significantly different from that of day 0.

To expand on the antigenic cartography findings identifying Abs to GII.4.1997 as key driving components of the vaccine-induced Ab response, we then asked whether the GII.4 Ab response generated after vaccination could be explained by activation of Abs to known GII.4 blockade epitopes A or F. We evaluated the ability of day 0, 7, 35, and 180 serum samples to block binding of mAbs to highly variable, surface-exposed blockade epitope A and sub-surface, conserved blockade epitope F using the blocking of binding (BOB) assay and GII.4.1997 and GII.4.2006b VLPs. GII.4.1997 and GII.4.2006b are antigenically distinct at evolving blockade epitope A (Fig. 8). Mouse mAb GII.4.1987.G1 and GII.4.2006.G2 [27] recognize epitope A of GII.4.1997 and GII.4.2006b, respectively. Mouse mAb GII.4.2002.G5 [32] recognizes the epitope F conserved among the GII.4 types. All serum samples remaining were tested for BOB. Pre-vaccination sera (*n* = 9) did not block binding of mAbs to epitope A or to epitope F to either VLP (Fig. 11). At day 7 (*n* = 7), significant broad BOB of epitope A and F mAbs was observed for both GII.4.1997 and GII.4.2006b, with significantly higher levels of inhibition noted against the GII.4.1997 strain for both epitopes (epitope A, 7-fold higher; epitope F, 5-fold higher). By day 35 (*n* = 8), significantly higher BOB titers to only epitope A of both strains remained. At day 180, titers (*n* = 8) to GII.4.1997 epitope A persisted but at levels not significantly different from baseline titers. These findings support the antigenic cartography analyses indicating that Ab to structures present in GII.4.1997 may be more persistent than Ab to other structures. Further, it suggests that the broad cross-reactive GII.4 Ab response targets residues either within, near, or regulating access to multiple epitopes, including epitopes A and F.

## Discussion

The success of the rotavirus vaccination campaign has elevated NoV to the main cause of acute viral gastroenteritis in infants and young children in the US and elsewhere, and, subsequently, has focused attention on the development of a NoV vaccine (reviewed in [36,50]). Here we provide evidence that a NoV VLP-based vaccine induced broadly reactive IgG and blockade Ab responses among antigenically diverse NoV VLPs, as has been shown for mice immunized with a multivalent NoV VLP vaccine [44] and humans infected with GI.1 [51]. Ab cross-reactivity included two GII.4 VLPs representing viral strains that did not circulate within the population before the study sample collection was completed, indicating that vaccination may protect against emergent GII.4 strains that are antigenically distinct from the vaccine components.

As NoV strains share common Ab epitopes, the Ab detection assays utilized here were optimized for specificity by limiting the amount of antigen. This approach allows detection of subtle differences in Ab affinities between closely related strains but decreases assay sensitivity, resulting in more samples registering below the limit of detection. Similarly, the high valency of PGM facilitates binding of many diverse NoV VLPs to a single, readily available substrate but also translates to increased serum titers needed for Ab blockade of VLP–ligand binding compared to the less valent synthetic carbohydrates frequently used [25,29]. Consequently, the magnitude and duration of Ab responses (GMTs and GMFRs) in these analyses were lower than those reported by other vaccine investigations, which used ∼10× more antigen in Ab detection assays and synthetic carbohydrates for blockade Ab assays [37]. The limiting amount of antigen likely also caused underestimation of the baseline differences between participants of secretor-negative and -positive phenotypes. Further, the VLPs used here, except GII.4C, were produced at 37°C from mammalian cell expression vectors, whereas the GII.4C VLPs and the vaccine VLPs were produced at a lower temperature in insect cells. How these differences in VLP production might affect particle structure and Ab binding is unclear, but antigenic differences have been described between NoV VLPs manufactured in the two different expression systems [32].

**Fig 12:**
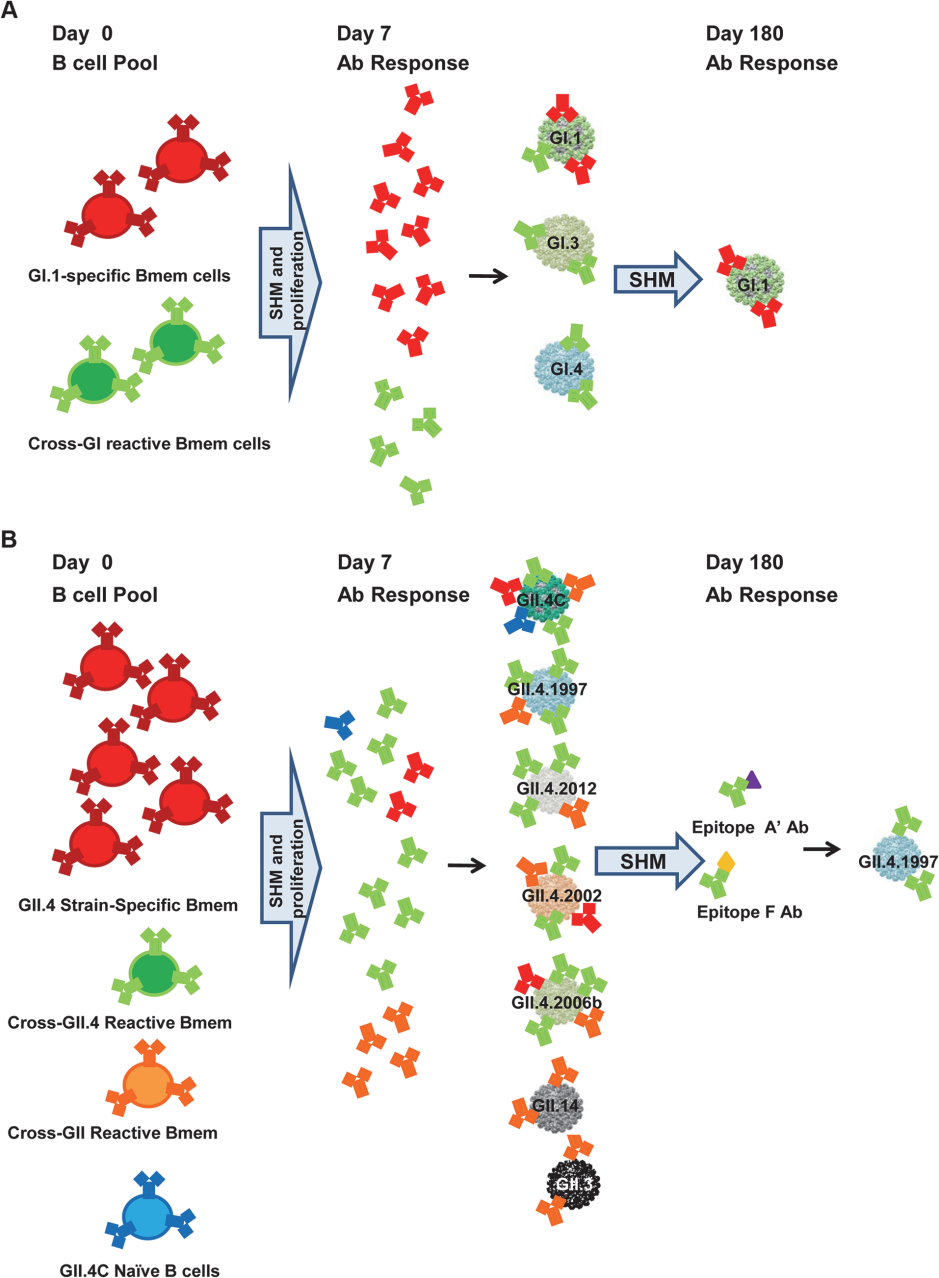
Proposed mechanisms for antibody responses induced by GI.1/GII.4C multivalent vaccine. At the time of vaccination, adult participants have a lifetime of NoV exposure history and a pool of NoV-reactive memory B cells (Bmem cells), both strain-specific and strain cross-reactive clones. Vaccination activates memory B cells to undergo SHM of the variable region of the Ab gene, to proliferate, and, for some cells, to differentiate into plasma cells secreting high-affinity Ab by day 7 post-vaccination. The GI.1 vaccine component elicits activation of both GI.1-specific memory B cells and memory B cells with specificity for shared GI epitopes, resulting in increased Ab to the panel of GI VLPs, but the strongest response to the homotypic GI.1 VLP because more blockade epitopes are unique to GI.1 than are shared across the GI VLPs. By day 180, low levels of GI.1-specific Ab persist (A). In comparison, the GII.4C vaccine component does not elicit a strong strain-specific response but could in theory activate memory B cells with GII.4.2002, GII.4.2006a, or GII.4.2006b specificity. However, the uniformity in the GII.4 VLP response across an antigenically diverse panel suggests that GII.4C preferentially activates memory B cells for conserved GII.4 epitopes and a smaller subset of memory B cells for a conserved GII epitope, resulting in more potent GII.4 and less potent GII blockade Ab production at day 7. The GII.4C Ab response continues to track with GII.4.2002 and GII.4.1997 through day 35, but by day 180, only GII.4.1997 Ab responses remain distinct, suggesting that the common GII.4 blockade epitopes recognized by the vaccine-induced Abs are most similar to sequences found in GII.4.1997, possibly because of extensive long-term immune focusing for this strain. Ab responses to at least two epitopes are maintained. Epitope F is a conserved GII.4 blockade epitope located sub-surface on the particle, and proposed epitope A′ is likely a surface-exposed blockade epitope physically near, overlapping, or within epitope A (B).

Pre-exposure history likely shaped the breadth and magnitude of the vaccine-induced blockade Ab cross-reactivity. Although a secretor-negative phenotype is associated with decreased risk of infection with some NoV strains [18,40], secretor status was not a primary driver of vaccine response, as secretor-negative participants responded similarly to secretor-positive participants for blockade Ab to the entire panel of VLPs, in concordance with in vitro [38] and in vivo [39–41] data indicating that some GI and GII NoV strains bind to secretor-negative carbohydrates and infect secretor-negative individuals. Further, as GII.4 strains commonly circulate, it is likely that participants in the study have a recent exposure history to GII.4, and this may have influenced the characteristics of the vaccine response. The increased ratio of blockade Ab to EIA-reactive IgG across GII.4 VLPs suggests that, likely as a result of previous exposure to GII.4 strains through natural infection, the GII.4C vaccine component response may be driven by activation of preexisting, cross-reactive memory B cells, as has been described for broadly neutralizing influenza Ab [52,53], while the GI.1 response may be driven by SHM and expansion of more strain-specific B cells (Fig. 12). Higher preexisting blockade Ab titers did not interfere with additional Ab production, but individuals with higher baseline titers tended to have lower fold increases, which may be indicative of a ceiling for maximum responses, as described for measles virus vector and some influenza A vaccinations [54,55]. Similarly, lack of an additional titer boost after a second vaccination <1 mo later is documented for influenza vaccinations, where the vaccine regimen achieves high levels of seroresponse and seroprotection (hemagglutination inhibition > 40) after only one dose [56,57], indicating that higher Ab titers correlate with a lower magnitude of response but not necessarily less protection from infection. Of note, in the influenza studies cited above, children and older adults had better Ab responses after two vaccine doses. Given that both of these demographics would be key candidates for a NoV vaccine, follow-up studies of different age cohorts will be necessary and are ongoing. Such studies will specifically address the kinetics and breadth of the blockade Ab response in children and may clarify the relationship between pre-exposure history and vaccination response.

At day 7 post-vaccination, Ab titers peak in magnitude and breadth of reactivity, corresponding with the timing of an expanded B cell clonal population [58]. This broad reactivity may be the result of the multivalency of the VLP vaccine, epitope presentation on a VLP compared to natural virus, or the difference in dose or route of antigen delivered by vaccination versus infection. Alternatively, it is possible that a broad blockade Ab response also occurs after natural infection at day 7, as this has not been tested. Although vaccination induced homotypic serum Ab responses at levels similar to or higher than natural infection in a challenge study [42], titers did not boost after a second immunization, agreeing with our findings. These data suggest one vaccine dose may induce maximum responses. Although, mechanistically, it is unclear why this would be the case in participants with lower preexisting titers, these findings may indicate that for the age group 18–49 y (characterized by extensive previous exposure history), more time may be needed between the first and second vaccination to allow titers to return to baseline and the Ab pool to mature and develop higher affinities. Alternately, serum titers may be decreasing because ASCs may be relocating to the mucosal induction sites (e.g., Peyer’s patches in the intestine), with a resulting boost in a responder B cell population at the site of future exposure to NoV. B cell and ASC migration to the gut has been shown to be critical for conferring protection against intestinal pathogens [59], and memory B cells from beta-7-integrin-deficient mice have been shown to exhibit a decreased capacity to protect against rotavirus infections, implying that gut homing plays an important role in protection against intestinal viruses [60]. To understand NoV immunity and protection from infection, direct measurement of mucosal Ab responses is needed, as mucosal IgA responses have been shown to correlate with protection in GI.1 virus challenge [18]. Challenge studies in vaccinated and non-vaccinated participants need to be done to address these fundamental questions.

The technique of antigenic cartography has been implemented by the World Health Organization to track influenza [49], as the power of cartography in sorting antigenic variants has been demonstrated for influenza [61], enterovirus [62], and NoV [63]. This technique has typically been used to assess the general relationships between variant viruses, using hyperimmune sera to assess antigenic similarities. Here we have slightly modified this approach to utilize variant viruses as probes to disentangle the complex immune responses following vaccination, while maintaining a well-developed analytical framework. This approach recapitulated many of the findings observed by our more traditional analyses of immune responses, and also allowed us to identify several more subtle differences in immune responses across this population of vaccine recipients. Here, IgG and blockade Ab responses to GI.1 are less distant from those to other GI VLPs compared to those to non-GI VLPs, but remain clearly separate from responses to GI.3 and GI.4. In contrast, IgG and blockade Ab responses cluster GII.4C close to the other GII.4 VLPs, while the distance between GII.4C and the other GII VLPs is greater. The distance, and thus antigenic divergence, between GII.4C and GII.4.1997 and GII.4.2002 is relatively close at day 7. By day 180, GII.4C and GII.4.2002 associate very closely with each other and cluster more closely to the other VLPs, and only GII.4.1997 remains distinct.

The strength of the blockade Ab response to GII.4.1997 and degree of cross-blockade among GII.4 VLPs is contrary to findings with mAbs and polyclonal serum collected from outbreaks and immunized mice. Pandemic GII.4 strains can be considered successive immune escape variants. The GII.4 VLPs studied here represent the antigenic diversity of the GII.4 outbreak strains that have circulated worldwide from 1995 to the present. Using human outbreak and immunized-mouse polyclonal sera, GII.4.1997 and GII.4.2002 have some cross-blockade activity between them and are each distinct from GII.4C at the identified GII.4 evolving blockade epitopes A, D, and E. However, GII.4.1997, GII.4.2002, and GII.4C are all blocked by mAbs that recognize epitope F, a conserved GII.4 blockade epitope [25,32], indicating a possible mechanism for cross-GII.4 blockade Ab activity, despite antigenic drift within the GII.4 strains.

Vaccination did lead to increased titers of Ab that interfered with binding of epitope F mAb to GII.4.1997 and GII.4.2006b, as measured by the BOB assay, supporting activation of cross-GII.4 memory B cells with vaccination. Epitope F is conserved but not identical within the GII.4 strains [25], explaining the difference in epitope F BOB potency between GII.4.1997 and GII.4.2006b. Epitope A is highly divergent between pandemic GII.4 strains; thus, it was an unlikely candidate to explain the broad GII.4 blockade Ab response identified here. However, minimizing steric hindrance as the explanation for these results, binding of mAb to epitope F or epitope A did not interfere with subsequent binding of the reciprocal epitope mAb to the immobilized VLP, indicating that Ab binding to one epitope does not necessarily prevent Ab binding at a second epitope [32,64].

Alternatively, the serum BOB to epitope A could be mediated by a rare class of cross-reactive epitope A Abs that are expressed post-vaccination because of the novel antigen that GII.4C represents. Because epitope A is the immunodominant epitope, NoV memory B cells should be numerically dominated by epitope A clones. If, as a chimeric-designed VLP, GII.4C has a unique epitope A, then vaccination could preferentially select for clones that recognize a conserved set of residues that are either part of epitope A or structurally near epitope A but are as yet undefined. Despite significant change within epitope A between GII.4 strains, epitope A does have a linear, conserved stretch of amino acids with demonstrated cross-GII.4 EIA binding with select mAbs [20,27], providing mechanistic support for the possibility of a broadly reactive GII.4 epitope A Ab response. Additional conserved epitope A residues may be identified as more mAbs are developed. The high degree of cross-GII.4 reactivity prevented us from using epitope-exchanged VLPs to specifically map GII.4 epitopes, as we have successfully done with mAbs and sera from infected individuals [25,27].

Both epitope mapping and antigenic cartography identified persistent responses to GII.4.1997, the first known pandemic NoV strain, after vaccination. As all study participants were at least 18 y old, it is highly likely that they were exposed to this first pandemic strain and retained memory B cells from that exposure. The preferential recall Ab response to GII.4.1997 after vaccination with an antigenically distinct VLP, coupled with lower Ab titers to the most recent pandemic strain, GII.4.2006b, all in the context of a broad cross-GII.4 blockade Ab response, suggests that the vaccine targets memory B cells specific for conserved epitopes that originally derived from naïve B cells specific for GII.4.1997 and were subsequently refined by SHM following repeated NoV exposure (Fig. 12). This response pattern, termed “antigenic seniority” has also been identified in influenza vaccinated participants [65]. Like original antigenic sin (OAS), the theory of antigenic seniority predicts that the highest affinity Ab responses will be to the strain primarily circulating during participants’ childhoods and that this first encounter will shape all immune responses to viral variants going forward. With each subsequent virus exposure, memory B cells undergo further maturation. Unlike OAS, antigenic seniority doesn’t predict that responses to the current virus will be negated by the response to the first virus, but instead that the Ab response will be lower in affinity for the most recent variant. Alternatively, vaccination, as opposed to infection, may produce Ab to conserved epitopes that either have a greater affinity for the epitope as it is presented in GII.4.1997 or have better access to the epitope in GII.4.1997. If this is true, titers to GII.4.1997 would predominate and may define the Ab response maximum described. It remains to be seen whether these same Ab response patterns develop with virus challenge. Sequencing and cloning of human Ab repertoires at pre-vaccination and early and late post-vaccination time points, coupled with investigation of the structures of Ab-bound VLPs, would directly address how the Ab response evolves. We further note that we did not see preferred recall of non-vaccine-strain responses to the rarely circulating GI NoVs. While this may be due to the overall similarities within the GI or the GII clusters, our expectation is that completely naïve immune responses should most match the GI.1 or GII.4C vaccine components, and not a non-vaccine-component VLP.

The primary limitation of this study is the incomplete understanding of the relationship between the in vitro blockade Ab assay and protection from infection and illness. In a vaccine challenge study with a GII.4.2002 challenge virus, vaccination did not significantly protect from infection, although participant-reported gastroenteritis symptoms were reduced and symptom severity was decreased. It is difficult to compare these findings to our prediction of potential cross-protection based on blockade Ab response because blockade Ab titers were not reported for those participants [42]. Further, the limited-antigen design of our experiments complicates prediction of protection from infection and understanding of the implications of the lack of titer boost after the second dose and rapid waning of the Ab titer. The diversity of the VLP panel studied is not all-inclusive, as new NoV strains are regularly identified from outbreak investigations. The VLPs used here were selected for their epidemiological importance, antigenic divergence, and availability. Although we cannot discount that Ab responses to additional genotypes may have been identified with a larger VLP panel, to our knowledge, this study characterizes blockade Ab responses to the most antigenically diverse panel of VLPs to date. Furthermore, our work here highlights in many ways the complex interactions between prior exposure, each participant’s ability to respond to variable vaccine components, and the relationships between tested VLPs. Future work assessing the impact of all of these variables will assist in the rational design of vaccines. Despite these limitations, we were able to utilize our molecular, immunological, and computational approaches to identify several important responses to NoV vaccination that allowed for cross-reactivity to other VLPs.

To be effective, any NoV vaccine must induce a protective immune response to novel, epitope-evolved GII.4 viruses. The data presented here suggest that the GII.4C vaccine component Ab response is likely driven by activation of preexisting cross-reactive memory B cells, resulting in cross-blockade Ab production and potential protection from new GII.4 strains not included in the vaccine. Given the considerable disease burden globally, these results provide sufficient optimism for continued human studies and vaccine development.

## Supporting Information

S1 Fig Mean EC_50_ IgG titer in vaccinated participants by secretor phenotype. Serum samples collected from participants who received the 50/50-μg VLP dose were assayed for IgG reactivity to a panel of GI (blue), GII.4 (grey), and non-GII.4 GII (green) VLPs and stratified by secretor (sec) phenotype. The seroresponse rate is the ratio of the number of participants with a ≥4-fold titer increase above day 0 titer compared to the total number of samples tested at day 0 for each VLP. Bolded values denote significantly different responses between the secretor phenotypes. Participants are ordered by day 0 GI.1-reactive IgG.
(DOCX)

S2 Fig Mean EC_50_ blockade antibody titer in vaccinated participants by secretor phenotype. Serum samples collected from participants who received the 50/50-μg VLP dose were assayed for blockade Ab to a panel of GI (blue), GII.4 (grey), and non-GII.4 GII (green) VLPs and stratified by secretor (sec) phenotype. The seroresponse rate is the ratio of the number of participants with a ≥4-fold titer increase above day 0 titer compared to the total number of samples tested at day 0 for each VLP. Bolded values denote significantly different responses between the secretor phenotypes.
(DOCX)

S3 Fig Mean EC_50_ IgG titer in vaccinated participants by individual. Serum samples collected from participants who received the 50/50-μg VLP dose were assayed for IgG reactivity to a panel of GI (blue), GII.4 (grey), and non-GII.4 GII (green) VLPs. Participants are listed in order of increasing day 0 IgG titer to GI.1. Bold values indicate a titer ≥4-fold increase over the titer at day 0. Participants are ordered by day 0 GI.1-reactive IgG. NA, not available (either due to missed follow-up sample collection or sample volume depletion); ND, not determined.
(XLSX)

S4 Fig Mean EC_50_ blockade antibody titer in vaccinated participants by individual. Serum samples collected from participants who received the 50/50-μg VLP dose were assayed for blockade Ab to a panel of GI (blue), GII.4 (grey), and non-GII.4 GII (green) VLPs. Participants are listed in order of increasing day 0 IgG titer to GI.1. Bold values indicate a titer ≥4-fold increase over the titer at day 0. NA, not available (either due to missed follow-up sample collection or sample volume depletion); ND, not determined.
(XLSX)

S5 Fig Ratio of blockade antibody to IgG by day and VLP among the group receiving the 50/50-μg VLP dose. The ratio of blockade Ab to IgG was determined for GI (blue), GII.4 (grey), and non-GII.4 GII (green) VLPs as a potential marker for Ab response maturity. ND, not determined.
(XLSX)

S1 Table Cartography distance values (*D*) by antibody, day, and virus-like particle. (XLSX)

## Abbreviations:

Ab: antibody
ASC: antibody-secreting cell
BOB: blocking of binding
EIA: enzyme immunoassay
GEE: generalized estimating equation
GMFR: geometric mean fold rise
GMT: geometric mean titer
HBGA: histoblood group antigen
mAb: monoclonal antibody
MDS: multi-dimensional scaling
NoV: norovirus
OAS: original antigenic sin
PBMC: peripheral blood mononuclear cell
PGM: porcine gastric mucin
SHM: somatic hypermutation
VLP: virus-like particle
