# Supplementary material for: Broad Blockade Antibody Responses in Human Volunteers after Immunization with a Multivalent Norovirus VLP Candidate Vaccine: Immunological Analyses from a Phase I Clinical Trial
Source: PLoS Med. 2015 Mar 24;12(3):e1001807. doi: 10.1371/journal.pmed.1001807 (PMC4371888; doi:10.1371/journal.pmed.1001807)
Supplement: S1 Fig — Serum samples collected from participants who received the 50/50-μg VLP dose were assayed for IgG reactivity to a panel of GI (blue), GII.4 (grey), and non-GII.4 GII (green) VLPs and stratified by secretor (sec) phenotype. The seroresponse rate is the ratio of the number of participants with a ≥4-fold titer increase above day 0 titer compared to the total number of samples tested at day 0 for each VLP. Bolded values denote significantly different responses between the secretor phenotypes. Participants are ordered by day 0 GI.1-reactive IgG. (DOCX) [file pmed.1001807.s001.docx]

**Figure S1. Mean Ec50 IgG titers in vaccinated participants by secretor phenotype.**

|  | **Day 0** | | **Day 7** | | **Day 21** | | **Day 35** | | **Day 180** | |
| --- | --- | --- | --- | --- | --- | --- | --- | --- | --- | --- |
|  | **Sec-** | **Sec+** | **Sec-** | **Sec+** | **Sec-** | **Sec+** | **Sec-** | **Sec+** | **Sec-** | **Sec+** |
| GI.1 |  |  |  |  |  |  |  |  |  |  |
| GMT (95% CI) | **50.0 ( - )** | **119.7**  **(64.5, 222.0)** | 910.3  (419.0, 1977.6) | 841.6  (264.1, 2682.0) | 781  (596.5, 1022.7) | 1007.9  (311.7, 3259.4) | 784.2  (499.3, 1231.7) | 1193  (447.6, 3180.0) | **173.0**  **(103.0, 290.4)** | **533.9**  **(235.6, 1210.0)** |
| GMFR (95% CI) |  |  | 18.2  (8.4, 39.6) | 7.0  (2.7, 18.6) | 15.6  (11.9, 20.5) | 8.4  (3.1, 23.0) | 15.7  (10.0, 24.6) | 10.0  (4.7, 20.9) | 3.5  (2.1, 5.8) | 4.5  (2.5, 7.9) |
| # Samples Tested  (# Matched to Day 0) | 4 | 6 | 4 (4) | 6 (6) | 4 (4) | 6 (6) | 4 (4) | 6 (6) | 3 (3) | 6 (6) |
| Seroresponse Rate |  |  | 4/4 | 4/6 | 4/4 | 5/6 | 4/4 | 6/6 | 1/3 | 4/6 |
| GI.3 |  |  |  |  |  |  |  |  |  |  |
| GMT (95% CI) | 97.9  (47.6, 201.0) | 154.9  (79.2, 302.8) | 1458.6  (425.0, 5006.6) | 726.7  (175.6, 3006.8) |  |  | 498.2  (237.3, 1046.0) | 518.8  (150.9, 1782.9) | 283.3  (92.2, 870.8) | 365.6  (83.8, 1595.0) |
| GMFR (95% CI) |  |  | 14.9 (4.9, 45.8) | 4.7 (1.3, 16.5) |  |  | 5.1 (3.2, 8.0) | 3.3 (1.1, 10.2) | 2.3 (0.7, 7.3) | 2.4 (0.8, 7.4) |
| # Samples Tested  (# Matched to Day 0) | 4 | 6 | 4 (4) | 6 (6) |  |  | 4 (4) | 6 (6) | 3 (3) | 6 (6) |
| Seroresponse Rate |  |  | 4/4 | 4/6 |  |  | 3/4 | 2/6 | 0/3 | 2/6 |
| GI.4 |  |  |  |  |  |  |  |  |  |  |
| GMT (95% CI) | **50.0 ( - )** | **99.8**  **(56.6, 176.0)** | 861.0  (184.5, 4017.6) | 436.0  (148.2, 1282.6) |  |  | 336.3  (73.9, 1530.2) | 329.8  (94.9, 1145.8) | 90.0  (25.3, 320.9) | 231.6  (94.8, 565.8) |
| GMFR (95% CI) |  |  | 17.2  (3.7, 80.4) | 4.4  (1.2, 15.7) |  |  | 6.7  (1.5, 30.6) | 3.3  (0.9, 11.9) | 1.8  (0.5, 6.4) | 2.3  (1.0, 5.5) |
| # Samples Tested  (# Matched to Day 0) | 4 | 6 | 4 (4) | 6 (6) |  |  | 4 (4) | 6 (6) | 3 (3) | 6 (6) |
| Seroresponse Rate |  |  | 4/4 | 3/6 |  |  | 3/4 | 2/6 | 0/3 | 1/6 |
| GII.4C |  |  |  |  |  |  |  |  |  |  |
| GMT (95% CI) | 50.0 ( - ) | 89.8  (33.8, 238.1) | 386.8  (142.3, 1051.6) | 486.2  (152.0, 1555.2) | 320.1  (111.3, 920.8) | 453.6  (182.5, 1127.3) | 290.3  (151.8, 555.0) | 345.4  (204.9, 582.1) | 123.9  (90.4, 169.8) | 152.0  (74.5, 310.2) |
| GMFR (95% CI) |  |  | 7.7  (2.8, 21.0) | 5.4  (1.0, 30.1) | 6.4  (2.2, 18.4) | 5.1  (1.1, 22.4) | 5.8  (3.0, 11.1) | 3.8  (1.1, 13.8) | 2.5  (1.8, 3.4) | 1.7  (0.5, 5.7) |
| # Samples Tested  (# Matched to Day 0) | 4 | 6 | 4 (4) | 6 (6) | 4 (4) | 6 (6) | 4 (4) | 6 (6) | 3 (3) | 6 (6) |
| Seroresponse Rate |  |  | 3/4 | 3/6 | 3/4 | 3/6 | 3/4 | 4/6 | 0/3 | 1/6 |
| GII.4.1997 |  |  |  |  |  |  |  |  |  |  |
| GMT (95% CI) | 60.3  (33.3, 109.3) | 144.0  (53.3, 389.4) | 445.0  (215.3, 919.8) | 602.2  (193.2, 1877.6) |  |  | 376.2  (178.1, 794.6) | 409.9  (169.2, 992.6) | 139.9  (82.1, 238.3) | 204.0  (89.2, 466.4) |
| GMFR (95% CI) |  |  | 7.4  (2.4, 22.4) | 4.2  (0.8, 21.1) |  |  | 6.2  (1.9, 20.9) | 2.8  (0.7, 12.1) | 2.2  (0.6, 8.2) | 1.4  (0.5, 4) |
| # Samples Tested  (# Matched to Day 0) | 4 | 6 | 4 (4) | 6 (6) |  |  | 4 (4) | 6 (6) | 3 (3) | 6 (6) |
| Seroresponse Rate |  |  | 3/4 | 2/6 |  |  | 3/4 | 2/6 | 0/3 | 1/6 |
| GII.4.2002 |  |  |  |  |  |  |  |  |  |  |
| GMT (95% CI) | 60.5  (33.0, 111.0) | 127.7  (47.1, 346.1) | 623.2  (206.8, 1878.2) | 416.5  (206.8, 838.9) |  |  | 267.6  (139.6, 512.9) | 297.5  (170.9, 518.2) | 143.7  (84.9, 243.2) | 176.7  (84.9, 367.9) |
| GMFR (95% CI) |  |  | 10.3  (2.3, 46.8) | 3.3  (1.1, 9.4) |  |  | 4.4  (1.6, 12.0) | 2.3  (1.0, 5.5) | 2.2  (0.5, 9.7) | 1.4  (0.7, 2.7) |
| # Samples Tested  (# Matched to Day 0) | 4 | 6 | 4 (4) | 6 (6) |  |  | 4 (4) | 6 (6) | 3 (3) | 6 (6) |
| Seroresponse Rate |  |  | 3/4 | 1/6 |  |  | 2/4 | 2/6 | 0/3 | 0/6 |
| GII.4.2006b |  |  |  |  |  |  |  |  |  |  |
| GMT (95% CI) | 50.0 ( - ) | 80.0  (32.7, 196.1) | 228.0  (115.8, 449) | 211.5  (103.4, 432.7) |  |  | 170.8  (109.6, 266.3) | 158.9  (99.1, 254.8) | 110.8  (74.4, 165.0) | 129.8  (52.1, 323.3) |
| GMFR (95% CI) |  |  | 4.6  (2.3, 9.0) | 2.6  (1.8, 3.9) |  |  | 3.4  (2.2, 5.3) | 2.0  (1.2, 3.3) | 2.2  (1.5, 3.3) | 1.6  (1.0, 2.8) |
| # Samples Tested  (# Matched to Day 0) | 4 | 6 | 4 (4) | 6 (6) |  |  | 4 (4) | 6 (6) | 3 (3) | 6 (6) |
| Seroresponse Rate |  |  | 3/4 | 1/6 |  |  | 1/4 | 0/6 | 0/3 | 0/6 |
| GII.2 |  |  |  |  |  |  |  |  |  |  |
| GMT (95% CI) | 138.6  (84.4, 227.7) | 206.4  (75.2, 566.5) | 399.2  (200.3, 795.4) | 220.0  (123.5, 391.7) |  |  | 319.2  (250.5, 406.7) | 248.6  (143.3, 431.2) | 159.8  (113.2, 225.6) | 147.2  (79.3, 273.3) |
| GMFR (95% CI) |  |  | **2.9**  **(0.9, 9.2)** | **1.1**  **(0.6, 1.8)** |  |  | **2.3**  **(1.4, 3.9)** | **1.2**  **(0.7, 2.0)** | 1.1  (0.7, 1.7) | 0.7  (0.4, 1.3) |
| # Samples Tested  (# Matched to Day 0) | 4 | 6 | 4 (4) | 6 (6) |  |  | 4 (4) | 6 (6) | 3 (3) | 6 (6) |
| Seroresponse Rate |  |  | 2/4 | 0/6 |  |  | 0/4 | 0/6 | 0/3 | 0/6 |
| GII.3 |  |  |  |  |  |  |  |  |  |  |
| GMT (95% CI) | 116.4  (40.4, 335.3) | 224.7  (83.7, 603.1) | 477.2  (370.0, 615.3) | 315.7  (173.3, 575.3) |  |  | 399.5  (239.4, 666.7) | 240.8  (104.4, 555.6) | 163.7  (99.7, 268.8) | 148  (82.4, 265.7) |
| GMFR (95% CI) |  |  | **4.1**  **(1.3, 12.5)** | **1.4**  **(0.9, 2.2)** |  |  | **3.4**  **(1.4, 8.5)** | **1.1**  **(0.7, 1.7)** | 1.0  (0.3, 6.3) | 0.7  (0.4, 1.2) |
| # Samples Tested  (# Matched to Day 0) | 4 | 6 | 4 (4) | 6 (6) |  |  | 4 (4) | 6 (6) | 3 (3) | 6 (6) |
| Seroresponse Rate |  |  | 2/4 | 0/6 |  |  | 2/4 | 0/6 | 0/3 | 0/6 |
| GII.14 |  |  |  |  |  |  |  |  |  |  |
| GMT (95% CI) | 60.3  (33.2, 109.6) | 128.7  (50.2, 330.0) | 329.4  (179.4, 605.0) | 161.1  (82.3, 315.3) |  |  | 232.2  (159.8, 337.6) | 159.9  (83.4, 306.5) | 105.5  (74.7, 149.0) | 104.4  (56.1, 194.1) |
| GMFR (95% CI) |  |  | **5.5**  **(3.8, 7.9)** | **1.3**  **(0.7, 2.3)** |  |  | **3.8**  **(2.0, 7.5)** | **1.2**  **(0.6, 2.6)** | **2.1**  **(1.5, 3.0)** | **0.8**  **(0.4, 1.6)** |
| # Samples Tested  (# Matched to Day 0) | 4 | 6 | 4 (4) | 6 (6) |  |  | 4 (4) | 6 (6) | 3 (3) | 6 (6) |
| Seroresponse Rate |  |  | 4/4 | 0/6 |  |  | 2/4 | 0/6 | 0/3 | 0/6 |
| GII.4.2012 |  |  |  |  |  |  |  |  |  |  |
| GMT (95% CI) | 60.3  (33.3, 109.2) | 83.5  (46.4, 150.4) | **767.3**  **(0.5, 1158113.5)** | **237.6**  **(141.2, 399.6)** | 227.9  (133.3, 389.9) | 220.8  (107.5, 453.6) | 209.8  (119.8, 367.7) | 164.2  (96.8, 278.6) | 130.4  (114.8, 148.1) | 105.2  (42.1, 262.8) |
| GMFR (95% CI) |  |  | 10.6  (0, 1831637.4) | 2.8  (1.3, 6.4) | 3.8  (2.6, 5.6) | 2.2  (1.2, 3.9) | 3.5  (1.5, 8.3) | 1.7  (1.0, 2.9) | 2.0  (0.7, 5.8) | 1.3  (0.8, 1.9) |
| # Samples Tested  (# Matched to Day 0) | 4 | 5 | 2 (2) | 5 (5) | 4 (4) | 6 (5) | 4 (4) | 6(5) | 3 (3) | 5(5) |
| Seroresponse Rate |  |  | 2/2 | 1/5 | 1/4 | 0/5 | 2/4 | 0/5 | 0/3 | 0/5 |
| GII.4.2006b.P.D302 |  |  |  |  |  |  |  |  |  |  |
| GMT (95% CI) | 105.3  (41.5, 267.6) | 102.2  (26.5, 393.6) | **512.5**  **(9.9, 26517.7)** | **177.4**  **(100.6, 313.1)** | 277.5  (120.1, 641.2) | 212.5  (105.2, 429.2) | 218.6  (53.8, 887.7) | 246.1  (110.8, 546.6) | 141.1  (116.4, 171.2) | 131.6  (97.3, 178.2) |
| GMFR (95% CI) |  |  | 3.4  (2.3, 5.2) | 1.6  (0.4, 6.9) | 2.6  (1.5, 4.7) | 1.8  (0.7, 4.3) | 2.1  (1.3, 3.6) | 1.9  (0.7, 5.1) | 1.4  (0.3, 6.7) | 1.3  (0.5, 3.3) |
| # Samples Tested  (# Matched to Day 0) | 4 | 4 | 2 (2) | 4 (3) | 4 (3) | 6 (4) | 3 (3) | 5 (4) | 3 (3) | 5(4) |
| Seroresponse Rate |  |  | 0/2 | 0/3 | 0/3 | 0/4 | 0/3 | 0/4 | 0/3 | 0/4 |
