# Supplementary material for: Broad Blockade Antibody Responses in Human Volunteers after Immunization with a Multivalent Norovirus VLP Candidate Vaccine: Immunological Analyses from a Phase I Clinical Trial
Source: PLoS Med. 2015 Mar 24;12(3):e1001807. doi: 10.1371/journal.pmed.1001807 (PMC4371888; doi:10.1371/journal.pmed.1001807)
Supplement: S2 Fig — Serum samples collected from participants who received the 50/50-μg VLP dose were assayed for blockade Ab to a panel of GI (blue), GII.4 (grey), and non-GII.4 GII (green) VLPs and stratified by secretor (sec) phenotype. The seroresponse rate is the ratio of the number of participants with a ≥4-fold titer increase above day 0 titer compared to the total number of samples tested at day 0 for each VLP. Bolded values denote significantly different responses between the secretor phenotypes. (DOCX) [file pmed.1001807.s002.docx]

**Figure S2. Mean EC_50_ blockade Ab titer in vaccinated participants by secretor phenotype.**

|  | **Day 0** | | **Day 7** | | **Day 21** | | **Day 35** | | **Day 180** | |
| --- | --- | --- | --- | --- | --- | --- | --- | --- | --- | --- |
|  | **Sec-** | **Sec+** | **Sec-** | **Sec+** | **Sec-** | **Sec+** | **Sec-** | **Sec+** | **Sec-** | **Sec+** |
| **GI.1** |  |  |  |  |  |  |  |  |  |  |
| GMT (95% CI) | 20.0 ( - ) | 20.0 ( - ) | 784.8  (293.1, 2101.6) | 520.4  (98.2, 2756.1) | 120.6  (23.5, 619.5) | 237.3  (39.4, 1429.2) | 181.5  (41.6, 792.3) | 299.7  (83.4, 1077.0) | 20.0 ( - ) | 63.1  (21.1, 188.9) |
| GMFR (95% CI) |  |  | 39.2  (14.7, 105.1) | 26.0  (4.9, 137.8) | 6.0  (1.2, 31) | 11.9  (2.0, 71.5) | 9.1  (2.1, 39.6) | 15.0  (4.2, 53.9) | 1.0 | 3.2 (1.1, 9.4) |
| # Samples Tested | 4 | 6 | 4 (4) | 6 (6) | 4 (4) | 6 (6) | 4 (4) | 6 (6) | 3 (3) | 6 (6) |
| (# Matched to Day 0) |  |  |  |  |  |  |  |  |  |  |
| Seroresponse Rate |  |  | 4/4 | 5/6 | 2/4 | 4/6 | 3/4 | 5/6 | 0/3 | 2/6 |
| GI.3 |  |  |  |  |  |  |  |  |  |  |
| GMT (95% CI) | 20.0 ( - ) | 29.5  (15.3, 56.6) | 344.2  (46.4, 2555.9) | 123.5  (17.8, 857.2) |  |  | 62.0  (7.6, 520.4) | 90.5  (25.2, 325.4) | 26.0  (7.7, 93.2) | 31.3  (15.1, 64.9) |
| GMFR (95% CI) |  |  | 17.2  (2.3, 127.8) | 4.2  (0.5, 35.0) |  |  | 3.1  (0.4, 26.0) | 3.1  (0.9, 10.4) | 1.3  (0.4, 4.7) | 1.1  (0.5, 2.3) |
| # Samples Tested | 4 | 6 | 4 (4) | 6 (6) |  |  | 4 (4) | 6 (6) | 3 (3) | 6 (6) |
| (# Matched to Day 0) |  |  |  |  |  |  |  |  |  |  |
| Seroresponse Rate |  |  | 4/4 | 2/6 |  |  | 2/4 | 2/6 | 0/3 | 1/6 |
| GI.4 |  |  |  |  |  |  |  |  |  |  |
| GMT (95% CI) | 20.0 ( - ) | 20.0 ( - ) | 101.8  (2.4, 4384.5) | 71.9  (12.4, 418.8) |  |  | 25.5  (11.8, 55.1) | 34.1  (17.3, 67.2) | 20.0 ( - ) | 23.1  16.0, 33.4) |
| GMFR (95% CI) |  |  | 5.1  (0.1, 219.2) | 3.6  (0.6, 20.9) |  |  | 1.3  0.6, 2.8) | 1.7  (0.9, 3.4) | 1.0 | 1.2  (0.8, 1.7) |
| # Samples Tested | 4 | 6 | 3 (3) | 5 (5) |  |  | 4 (4) | 6 (6) | 3 (3) | 6 (6) |
| (# Matched to Day 0) |  |  |  |  |  |  |  |  |  |  |
| Seroresponse Rate |  |  | 2/3 | 2/5 |  |  | 0/4 | 1/6 | 0/3 | 0/6 |
| GII.4C |  |  |  |  |  |  |  |  |  |  |
| GMT (95% CI) | 20.0 ( - ) | 37.7  (12.6, 113.0) | 1401.8  (1108.6, 1772.4) | 737.4  (137.5, 3955.8) | 449.5  (272.6, 741.2) | 406.8  (138.0, 1199.7) | 192.9  (104.3, 356.9) | 219.7  (91.1, 529.5) | 28.0  (6.6, 119.4) | 93.1  (30.0, 288.4) |
| GMFR (95% CI) |  |  | 70.1 (55.4, 88.6) | 19.5 (1.8, 213.8) | 22.5 (13.6, 37.1) | 10.8 (1.9, 61.9) | 9.6 (5.2, 17.8) | 5.8 (1.3, 26.3) | 1.4 (0.3, 6.0) | 2.5 (0.6, 10.3) |
| # Samples Tested | 10 | 10 | 4 (4) | 6 (6) | 4 (4) | 6 (6) | 4 (4) | 6 (6) | 3 (3) | 6 (6) |
| (# Matched to Day 0) |  |  |  |  |  |  |  |  |  |  |
| Seroresponse Rate |  |  | 4/4 | 4/6 | 4/4 | 4/6 | 4/4 | 4/6 | 0/3 | 2/6 |
| GII.4.1997 |  |  |  |  |  |  |  |  |  |  |
| GMT (95% CI) | 20.0 ( - ) | 55.9  (9.6, 326.5) | 272.2  (12.2, 6083.4) | 1649.4  (132.8, 20491.6) |  |  | 60.3  (8.2, 440.4) | 356.9  (67.0, 1900.1) | 28.6  (6.1, 134.4) | 193.8  (28.9, 1298.7) |
| GMFR (95% CI) |  |  | 13.6  (0.6, 304.2) | 29.5  (1.7, 508.6) |  |  | 3  (0.4, 22.0) | 6.4  (1.0, 42.8) | 1.4  (0.3, 6.7) | 3.5  (0.6, 19.1) |
| # Samples Tested | 4 | 6 | 4 (4) | 6 (6) |  |  | 4 (4) | 6 (6) | 3 (3) | 6 (6) |
| (# Matched to Day 0) |  |  |  |  |  |  |  |  |  |  |
| Seroresponse Rate |  |  | 4/4 | 4/6 |  |  | 1/4 | 3/6 | 0/3 | 2/6 |
| GII.4.2002 |  |  |  |  |  |  |  |  |  |  |
| GMT (95% CI) | 31.5  (7.4, 134.5) | 88.2  (20.4, 381.7) | 312.5  (18.1, 5400.7) | 1138.7  (112.3, 11544.7) |  |  | 70.2  (3.7, 1348.5) | 385.6  (50.5, 2945.7) | 26.1  (8.3, 81.6) | 93.9  (26.2, 337.0) |
| GMFR (95% CI) |  |  | 9.9  (2.4, 40.9) | 12.9  (0.5, 330.5) |  |  | 2.2  (0.4, 11.2) | 4.4  (0.4, 54.1) | 0.7  (0.2, 3.1) | 1.1  (0.3, 3.3) |
| # Samples Tested | 4 | 6 | 4 (4) | 6 (6) |  |  | 4 (4) | 6 (6) | 3 (3) | 6 (6) |
| (# Matched to Day 0) |  |  |  |  |  |  |  |  |  |  |
| Seroresponse Rate |  |  | 4/4 | 4/6 |  |  | 1/4 | 3/6 | 0/3 | 0/6 |
| GII.4.2006b |  |  |  |  |  |  |  |  |  |  |
| GMT (95% CI) | 20.0 ( - ) | 65.5  (15.4, 278.4) | 183.0  (6.7, 4967.7) | 635.5  (94.1, 4290.7) |  |  | 55.4  (4.3, 713.3) | 310.9  (101.8, 949.4) | 20.0 ( - ) | 65.8  (18.8, 230.7) |
| GMFR (95% CI) |  |  | 9.2  (0.3, 248.4) | 9.7  (0.6, 170.7) |  |  | 2.8  (0.2, 35.7) | 4.7  (0.6, 35.1) | 1.0 | 1.0  (0.2, 4.9) |
| # Samples Tested | 4 | 6 | 4 (4) | 6 (6) |  |  | 4 (4) | 6 (6) | 3 (3) | 6 (6) |
| (# Matched to Day 0) |  |  |  |  |  |  |  |  |  |  |
| Seroresponse Rate |  |  | 3/4 | 3/6 |  |  | 1/4 | 3/6 | 0/3 | 1/6 |
| GII.3 |  |  |  |  |  |  |  |  |  |  |
| GMT (95% CI) | 39.2  (11.4, 135.2) | 89.4  (33.7, 237.3) | 670.7  (0.0, >30,000.0) | 320.1  (123.0, 833.3) |  |  | 78.9  (49.5, 125.7) | 108.2  (43.0, 272.6) | 69.2  (37.1, 129.3) | 65.8  (24.2, 179.0) |
| GMFR (95% CI) |  |  | 8.7  (0.0, >30,000.0) | 4.6  (0.9, 22.8) |  |  | 2.0  (0.8, 5.3) | 1.2  (0.7, 2.0) | 1.4  (0.4, 5.2) | 0.7  (0.2, 2.7) |
| # Samples Tested | 4 | 6 | 2 (2) | 5 (5) |  |  | 4 (4) | 6 (6) | 3 (3) | 6 (6) |
| (# Matched to Day 0) |  |  |  |  |  |  |  |  |  |  |
| Seroresponse Rate |  |  | 1/2 | 2/5 |  |  | 1/4 | 0/6 | 0/3 | 0/6 |
| GII.14 |  |  |  |  |  |  |  |  |  |  |
| GMT (95% CI) | 20.0 ( - ) | 43.1  (17.3, 107.6) | 193.1  (0.5, 71532.5) | 665.7  (164.5, 2693.4) |  |  | **20.0 ( - )** | **89.6**  **(25.0, 320.5)** | 20.0 ( - ) | 58.9  (23.3, 149.1) |
| GMFR (95% CI) |  |  | 9.7  (0.0, 3576.6) | 16.7  (1.3, 213.7) |  |  | 1.0 | 2.1  (0.4, 9.6) | 1.0 | 1.0  (0.4, 4.2) |
| # Samples Tested | 4 | 6 | 2 (2) | 5 (5) |  |  | 4 (4) | 6 (6) | 3 (3) | 6 (6) |
| (# Matched to Day 0) |  |  |  |  |  |  |  |  |  |  |
| Seroresponse Rate |  |  | 2/2 | 3/5 |  |  | 0/4 | 2/6 | 0/3 | 1/6 |
| GII.4.2012 |  |  |  |  |  |  |  |  |  |  |
| GMT (95% CI) | 20.0 ( - ) | 52.8  (19.3, 144.7) | **48.9**  **(23.8, 100.4)** | **1436.5**  **(330.2, 6249.4)** | 139.2  (7.2, 2700.5) | 653.9  (92.7, 4611.2) | 44.5  (9.1, 217.2) | 213.0  (50.1, 905.8) | 20.0 ( - ) | 80.0  (14.8, 432.3) |
| GMFR (95% CI) |  |  | 2.4  (1.2, 5.0) | 26.6  (1.7, 418.1) | 7.0  (0.4, 135.0) | 12.4  (1.2, 128.4) | 2.2  (0.5, 10.9) | 4.0  (0.7, 23.2) | 1.0 | 1.5  (0.4, 5.7) |
| # Samples Tested | 4 | 6 | 2 (2) | 5 (5) | 4 (4) | 6 (6) | 4 (4) | 6 (6) | 3 (3) | 5 (5) |
| (# Matched to Day 0) |  |  |  |  |  |  |  |  |  |  |
| Seroresponse Rate |  |  | 0/2 | 4/5 | 2/4 | 4/6 | 1/4 | 3/6 | 0/3 | 1/5 |
| GII.4.2006b.P.D302 |  |  |  |  |  |  |  |  |  |  |
| GMT (95% CI) | 20.0 ( - ) | 57.7  (6.5, 512.9) | **45.8**  **(0.0, 1700908.5)** | **1755**  **(329.1, 9358.4)** | 222.2  (5.7, 8702.1) | 733.1  (168.8, 3183.3) | 60.6  (0.6, 6540.9) | 218.1  (47.6, 998.1) | 20.0 ( - ) | 78.9  (14.1, 440.0) |
| GMFR (95% CI) |  |  | 2.3  (0.0, 85045.4) | 28.9  (0.1, 11538.9) | 11.1  (0.3, 435.1) | 26  (4.0, 169.8) | 3.0  (0.0, 327.0) | 4.9  (0.3, 77.4) | 1.0 | 1.4  (0.4, 4.9) |
| # Samples Tested | 4 | 4 | 2 (2) | 4 (3) | 4 (4) | 6 (4) | 3 (3) | 5 (3) | 3 (3) | 5 (4) |
| (# Matched to Day 0) |  |  |  |  |  |  |  |  |  |  |
| Seroresponse Rate |  |  | 1/2 | 2/3 | 3/4 | 4/4 | 1/3 | 2/3 | 0/3 | 1/4 |
